# The RelB-BLNK Axis Determines Cellular Response to a Novel Redox-Active Agent Betamethasone during Radiation Therapy in Prostate Cancer

**DOI:** 10.3390/ijms23126409

**Published:** 2022-06-08

**Authors:** Luksana Chaiswing, Fangfang Xu, Yanming Zhao, Jon Thorson, Chi Wang, Daheng He, Jinpeng Lu, Sally R. Ellingson, Weixiong Zhong, Kristy Meyer, Wei Luo, William St. Clair, Daret St. Clair

**Affiliations:** 1 Department of Toxicology and Cancer Biology, University of Kentucky, 452 Health Sciences Research Building, Lexington, KY 40536, USA; ffang2@uky.edu (F.X.); yzhao@uky.edu (Y.Z.); 2 Center for Pharmaceutical Research and Innovation, Lexington, KY 40536, USA; jsthorson@uky.edu; 3 College of Pharmacy, Pharmaceutical Sciences Department, University of Kentucky, Lexington, KY 40536, USA; 4 Markey Biostatistics and Bioinformatics Shared Resource Facility, University of Kentucky, Lexington, KY 40536, USA; chi.wang@uky.edu (C.W.); daheng.he@uky.edu (D.H.); jinpeng.liu@uky.edu (J.L.); sel228@uky.edu (S.R.E.); 5 Department of Pathology and Laboratory Medicine, University of Wisconsin, Madison, WI 53705, USA; wzhong3@wisc.edu (W.Z.); kristymeyer@wisc.edu (K.M.); 6 Department of Radiation Medicine, University of Kentucky, Lexington, KY 40536, USA; wei.luo@uky.edu (W.L.); stclair@email.uky.edu (W.S.C.)

**Keywords:** redox state, RelB, BLNK, betamethasone, radiation

## Abstract

Aberrant levels of reactive oxygen species (ROS) are potential mechanisms that contribute to both cancer therapy efficacy and the side effects of cancer treatment. Upregulation of the non-canonical redox-sensitive NF-kB family member, RelB, confers radioresistance in prostate cancer (PCa). We screened FDA-approved compounds and identified betamethasone (BET) as a drug that increases hydrogen peroxide levels in vitro and protects non-PCa tissues/cells while also enhancing radiation killing of PCa tissues/cells, both in vitro and in vivo. Significantly, BET increases ROS levels and exerts different effects on RelB expression in normal cells and PCa cells. BET induces protein expression of RelB and RelB target genes, including the primary antioxidant enzyme, manganese superoxide dismutase (MnSOD), in normal cells, while it suppresses protein expression of RelB and MnSOD in LNCaP cells and PC3 cells. RNA sequencing analysis identifies B-cell linker protein (BLNK) as a novel RelB complementary partner that BET differentially regulates in normal cells and PCa cells. RelB and BLNK are upregulated and correlate with the aggressiveness of PCa in human samples. The RelB-BLNK axis translocates to the nuclear compartment to activate MnSOD protein expression. BET promotes the RelB-BLNK axis in normal cells but suppresses the RelB-BLNK axis in PCa cells. Targeted disruptions of RelB-BLNK expressions mitigate the radioprotective effect of BET on normal cells and the radiosensitizing effect of BET on PCa cells. Our study identified a novel RelB complementary partner and reveals a complex redox-mediated mechanism showing that the RelB-BLNK axis, at least in part, triggers differential responses to the redox-active agent BET by stimulating adaptive responses in normal cells but pushing PCa cells into oxidative stress overload.

## 1. Introduction

Prostate cancer (PCa) is the second leading cause of cancer deaths in males in the United States [1]. While radiation therapy (RT) is important to control the growth of cancer, it presents the significant risk of increasing unwanted side effects, including injury to normal tissues. Given that the incidence of PCa accounts for more than 10% of all cancer cases in the U.S., novel therapeutic approaches aimed at protecting against normal tissue injury while also increasing RT efficacy are urgently needed. The development of such approaches would have a major impact on PCa control and the quality of life of PCa survivors [2].

The generation of reactive oxygen species (ROS) accounts for nearly 50% of cancer treatments [3,4]. Low-to-intermediate levels of ROS stimulate growth and survival, whereas high levels of ROS stimulate death pathways [2,5,6,7]. Because the redox state of cancer is rewired, therapeutic approaches that use prooxidants to both push tumor cells into oxidative stress overload and stimulate adaptive responses in normal cells have been proposed as efficacious ways to selectively enhance the killing of cancer cells but not normal cells [8,9].

ROS have been implicated as second messengers for gene expression regulation. The NF-kB family is a transcription factor that is particularly sensitive to cellular redox changes. NF-kB exists in an inactive form in the cytoplasm, bound to inhibitory I B proteins. The effect of NF-kB activation on its target gene depends on the structure and accessibility of NF-kB binding sites in the promoter/enhancer region of the target gene. p50/p65 (NF-kB1/RelA) heterodimers and p50 homodimers are the most commonly investigated dimers in the NF-kB1 signaling pathway [10]. In comparison, the non-canonical dimer p52/RelB is understudied in tumorigenesis and cancer therapy [11,12]. Mechanistically, the activation of IKKα and the subsequent degradation of p100 lead to the recruitment of RelB and its adaptor proteins, which induces the formation of RelB dimers with other members of the NF-kB family, including p52, p50, and RelA [13,14]. When RelB binds to a promoter of a downstream target, its partners or regulators can be activated, which subsequently triggers intracellular signaling cascades that promote the aggressive growth of cancer cells. Our previous studies have demonstrated that overexpression of RelB protects PCa cells against RT and that knockdown of RelB sensitizes PCa to RT [15,16,17]. We also have shown that RelB expression increases tumorigenicity in a mouse PCa xenograft tumor model [18].

In this study, we tested the hypothesis that a mild prooxidant would stimulate adaptive responses in normal prostate during RT but push PCa cells into oxidative stress overload. To test this concept in an unbiased manner, we screened a library of FDA-approved drugs that exert opposite effects in normal and PCa cells and increase hydrogen peroxide (H_2_O_2_) in both normal and cancer cells. The criteria for identification of these compounds were that: (1) They protect non-cancer cells against RT-induced cytotoxicity; (2) They kill PCa cells; (3) They increase H_2_O_2_ level in PCa cells and non-cancer cells. We identified and verified that a steroid, betamethasone (BET), not only has all the required properties, but it also suppresses RelB expression in PCa cells and induces expression of RelB in non-cancer cells. Significantly, we identified the B-cell linker protein, BLNK, a protein essential for normal B-cell development [19], as a novel RelB complementary partner. The RelB-BLNK axis may serve as a novel target to address frequent therapeutic failures and prevent RT-induced normal tissue injury.

## 2. Results

### 2.1. Indentified BET as a Drug That Promotes Viability of Normal Cells and Induces Mortality of PCa Cells via H_2_O_2_ Production

**Figure 1:**
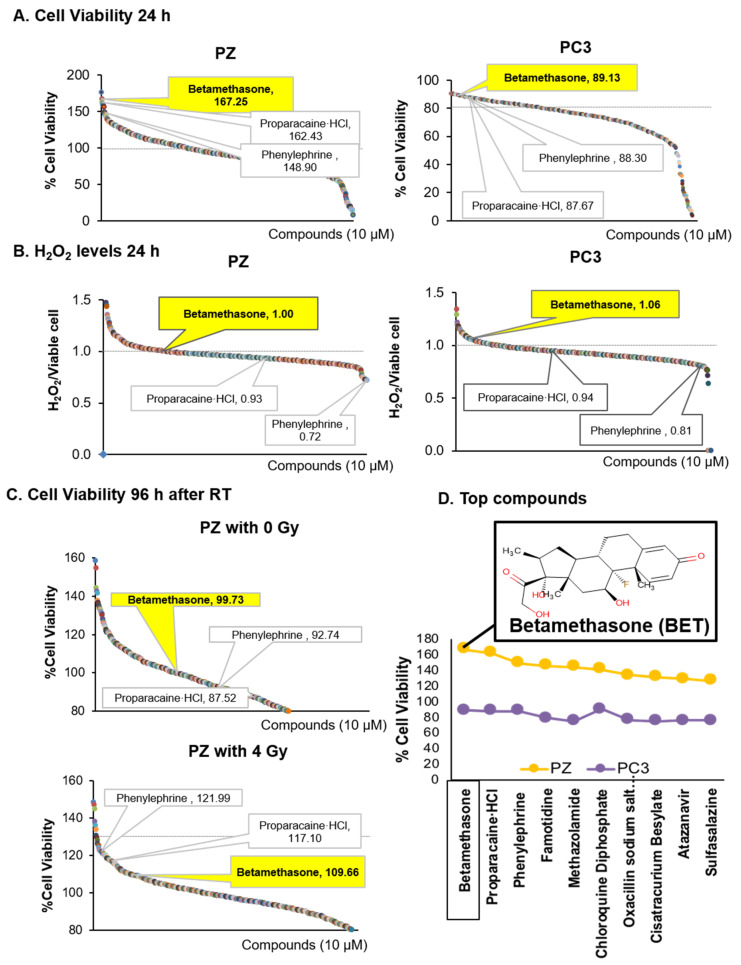
Library screening of FDA-approved drugs for compounds that induce PCa cell death while increasing non-cancer prostate cell viability through mediation of H_2_O_2_ production. PC3 cells and PZ cells were treated with library FDA-approved drugs for 24 h. (**A**) Cell viability. (**B**) Extracellular H_2_O_2_ based on Amplex Red assay. (**C**) Cell viability after drugs and radiation (4 Gy) treatment. Duplicate wells were tested per compound. (**D**) Drug candidates were chosen from a 786-compound library based on H_2_O_2_ production and the ability to exert opposite cytotoxic effects on non-cancer and cancer cells. One dot represents one compound. Please see supplementary excel file for details of each compound.

**Figure 2:**
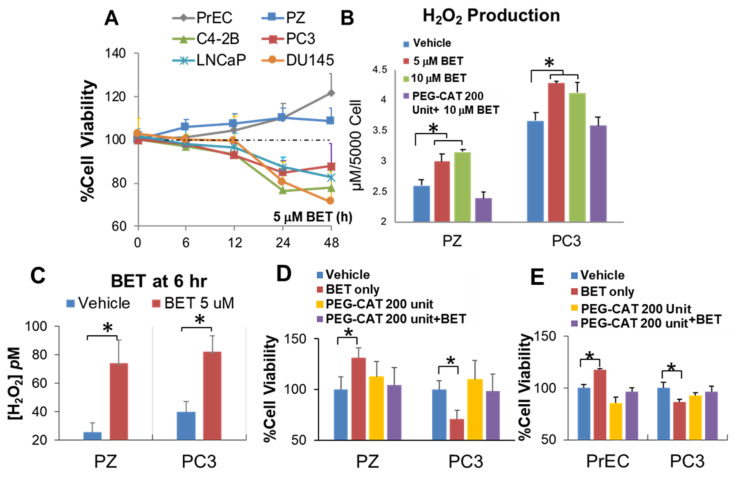
BET induces PCa cell death while increasing non-cancer prostate cell viability through mediation of H_2_O_2_ production. Cells were treated with 5 µM BET at various time points. (**A**) Cell viability based on MTT assay. (**B**) Extracellular H_2_O_2_ production (µM) based on Amplex Red method (24 h). (**C**) Intracellular H_2_O_2_ production was measured using the aminotriazole-(3-AT)-mediated inactivation of CAT method (6 h). (**D**,**E**) Cell viability after treatment with PEG-CAT prior to BET. * *p*-value ≤ 0.05 when compared with vehicle. *n* = 3.

To identify drugs that might be repurposed to protect non-cancer cells from RT off-target injury while enhancing the killing effect of RT, we used an unbiased, high-throughput drug screening approach. The MTT assay was used to measure cell viability in a 96-well format. Two thousand cells per well were incubated with drugs for 24 h. The DMSO tolerance assay indicated that the DMSO used in the screen did not significantly affect cell viability (Supplementary Figure S1). Data were normalized as the percentage of cell viability relative to DMSO control on each plate. A total of 786 drugs were tested. On the primary screen, compounds that reduced PC3 cell viability (less than 100%, 707 out of 786 drugs) while increasing immortalized prostate epithelial PZ-HPV-7 (PZ) cell viability (more than 100%, 263 out of 786 drugs), with the differences in percentage of cell viability between PZ cells and PC3 cells being greater than 50%, were considered to be positive (41 out of 786 drugs) (Figure 1A). On the second screen, two thousand cells per well were incubated with drugs for 24 h. H_2_O_2_ was then measured using an Amplex Red kit. The compounds that induced moderate levels of H_2_O_2_ production more than one-fold (indicating an increase in H_2_O_2_ production) in both PZ cells (120 out of 786 drugs) and PC3 cells (120 out of 786 drugs) were considered to be positive (Figure 1B). The ability of these compounds to inhibit PC3 cell viability was associated with a high level of H_2_O_2_ production (Supplementary Figure S1). Following the second screening, PZ cells were treated with drugs and RT (4 Gray (Gy)), and the MTT assay was employed after 96 h to identify compounds that increase PZ cell viability > 110% after RT treatment (88 out of 786 drugs, Top 10%) (Figure 1C). All three screenings identified at least 10 potential candidates; among these candidates, BET had the greatest impact: BET increased PZ cell viability, caused PC3 cell death, activated H_2_O_2_ production in PC3 cells, and protected PZ cells from RT-induced injury (Figure 1D). For validation, BET was tested with androgen-sensitive PCa cells, LNCaP and LNCaP-C4-2B (C4-2B), and androgen-independent PCa cells, PC3 and DU145. As shown in Figure 2A, BET decreased the cell viability of all PCa cells tested. The ability of BET to increase non-cancer cell viability was also confirmed in normal human prostate epithelial (PrEC) cells. Using fluorometric measurement of non-permeable Amplex Red, the basal level of H_2_O_2_ was significantly higher in PC3 cells than in PZ cells (Figure 2A). A quantification analysis showed that BET caused a significant increase in H_2_O_2_ in both PC3 and PZ cells, with a higher total H_2_O_2_ concentration in PC3 cells (Figure 2B,C). Treatment with Polyethylene glycol–Catalase (PEG-CAT) prior to BET treatment suppressed H_2_O_2_ levels to the basal level and reversed BET effects, including the inhibition of PC3 cell viability and the promotion of normal prostate epithelial cell viability in PZ cells and PrEC cells (Figure 2D,E). These data support that ROS, at least in part, mediate the effects of BET. Based on these validations, BET was used as a prototype redox-active drug in subsequent studies.

### 2.2. BET Acts as Radioprotector for Normal Tissues While Enhancing RT Efficacy in PCa In Vitro and In Vivo

**Figure 3:**
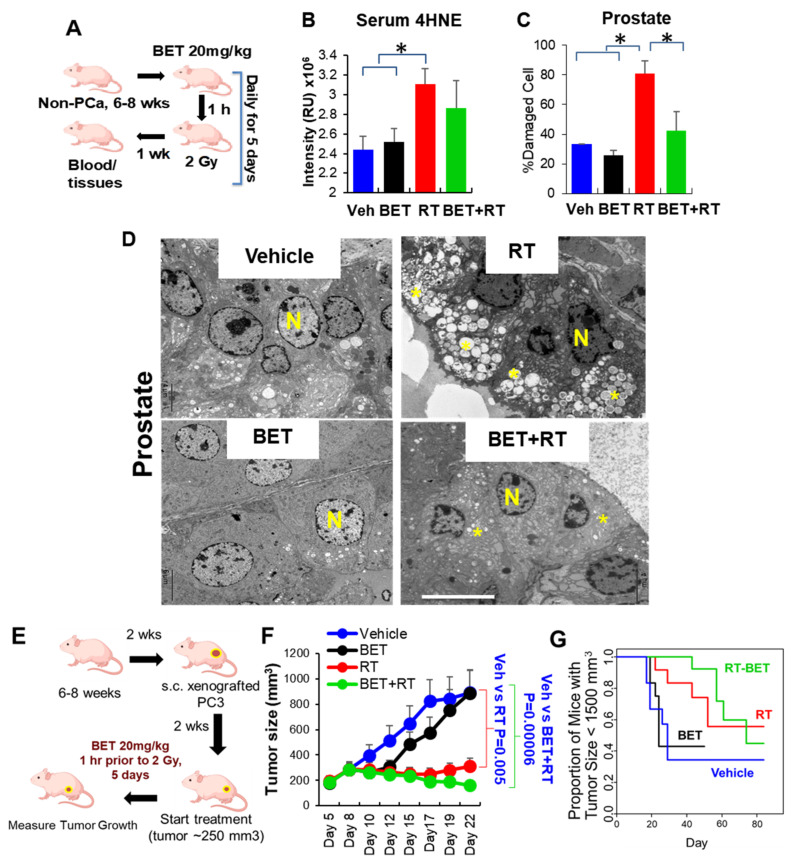
Repurposing BET as a radioprotector for normal tissues while enhancing radiation efficacy for PCa in vivo. (**A**) Non-cancer nude mice were I.P. with BET alone or RT (2 Gy) 1 h prior, daily, for 5 days. Prostate, rectum, and blood were collected 1 week after treatment was completed. *n* = 5/group. (**B**) 4HNE protein adducts in serum. RU = Relative arbitrary unit. (**C**) Ultrastructural analysis of damaged prostate. (**D**) Representative photographs of damaged prostate. *n* = Nucleus. Star = Damaged mitochondria as indicated with loss of cristae and vacuolization of mitochondria. Bar = 6 µm. (**E**) PC3 xenograft tumor mice were I.P. with BET alone or RT (2 Gy) 1 h prior, daily, for 5 days. *n* = 10/group. (**F**) PC3 tumor size was measured every 2–3 days. (**G**) Survival fraction of PC3 xenograft tumor mice. * *p*-value ≤ 0.05 when compared to vehicle.

To determine whether the differential effect of BET on normal cells prevents RT-induced cell death, PZ cells were treated with BET (1 to 20 µM) and RT (0 to 6 Gy). A colony survival assay was performed, and D0 (radiation dose that reduces the surviving number of cells to 37%) was calculated from a linear–quadratic curve survival model [20,21]. Treatment with a lower D0 value after treatments implies a sensitizing effect, compared to a higher D0 value. As shown in Table 1, the combined effects of BET and RT, as indicated by D0 in PZ cells, increased from 4.3 Gy to 4.7 Gy compared to RT alone, indicating a protective effect. Interestingly, D0 in PCa cells treated with a combination of BET and RT decreased from 3.2 Gy to 2.6 Gy in PC3 cells, from 3.2 Gy to 2.9 Gy in DU145 cells, and from 1.8 Gy to 1.65 Gy in LN-C42B cells, compared to RT alone, suggesting an additional killing effect. The percent survival curve (Supplementary Figure S2) after combination treatment with BET (1 to 20 µM) and radiation (0 to 6 Gy) confirms the killing effect of BET on PC3 cells without killing PZ cells. To further test whether BET exerts a differential effect on normal prostate tissues and PCa, the normal prostate of non-tumor mice was locally exposed daily to RT (2 Gy in five fractions, total of 10 Gy) (Figure 3A). One week after treatment, the serum levels of 4 hydroxynonenal (4HNE)-adducted protein, a marker of oxidative stress, were significantly increased in mice that received only RT and significantly reduced in mice that received BET prior to RT (Figure 3B), indicating that BET prevents normal prostate tissue from RT-induced injury. Importantly, an ultrastructural examination by transmission electron microscopy (EM) validated that injury of prostate tissues occurred in the RT group, but this injury was reduced by pretreatment with BET (Figure 3C,D). BET also reduced RT-induced rectal injury and protected the damage up to 4 weeks after treatment (Supplementary Figure S3). If BET is to be beneficial in clinical settings, it should not reduce the efficacy of RT. Thus, we tested the effect of BET on PC3 tumor growth in animals (Figure 3E). As expected, BET did not reduce RT efficacy, and although statistical significance was not achieved, BET provided an additional killing effect for RT as indicated by a decrease in tumor size up to 22 days (Figure 3F; after 22 days, some mice in the vehicle group and the BET group were euthanized due to their tumor size reaching 1500 mm^3^). The number of mice that survived post-treatment (with tumor size < 1500 mm^3^) were counted, and the survival fraction of tumor mice was calculated. Although it did not reach statistically significant, RT treatment alone slightly promoted the mice’s survival when compared to vehicle treatment (Figure 3G), whereas the BET+RT combination treatment statistically significantly promoted the mice’s survival when compared to vehicle treatment (*p* = 0.03). The tumor size of each mouse is shown in Supplementary Figure S4.

### 2.3. BET Inversely Regulates RelB Expression in Non-Cancer Cells and PCa 

**Figure 4:**
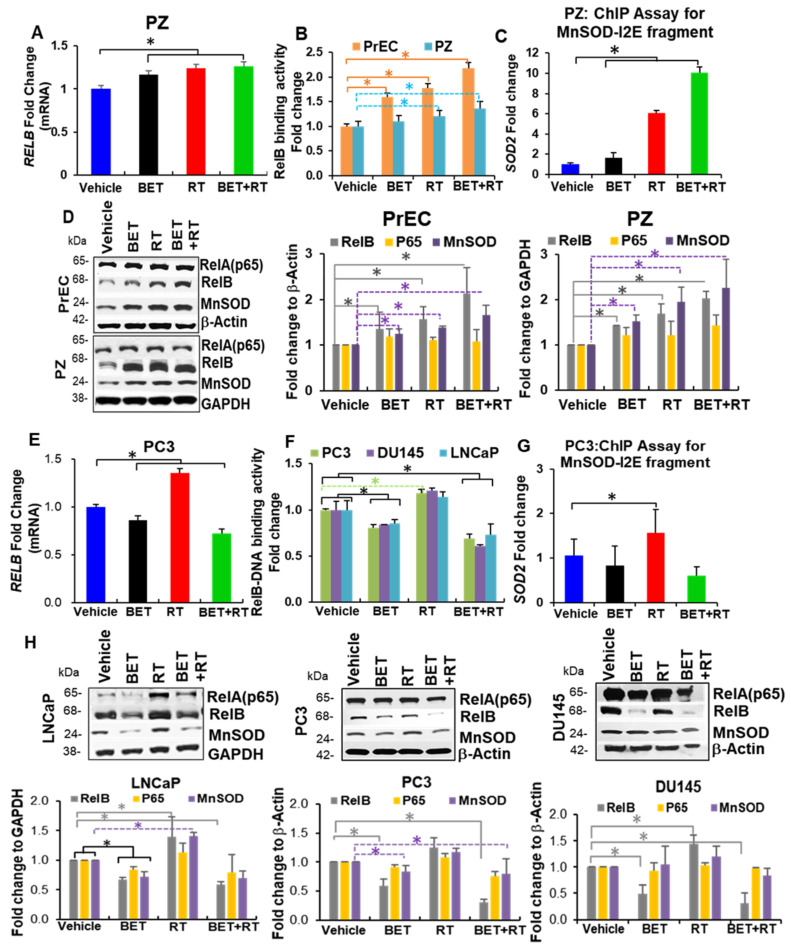
RelB expression determines radioprotector effect of BET in non-PCa cells and radio-killing effect of BET. PZ cells were treated with BET and/or RT (2 Gy) for 24 h. NF-kB transcription factor family expression levels and function were measured. (**A**) RT-PCR of RelB. (**B**) RelB binding activity. (**C**) Chip assay with RelB antibody to I2E promoter of MnSOD. (**D**) Representative Western blots and quantitative analysis of protein expression for PrEC cells and PZ cells. * *p*-value ≤ 0.05 when compared with vehicle. PCa cells (LNCaP, PC3, DU145) were treated with either BET and/or RT (2 Gy) for 24 h. NF-kB transcription factor family expression levels and function were measured. (**E**) RT-PCR of RelB of PC3 cells. (**F**) RelB binding activity of PCa cells. (**G**) Chip assay with RelB antibody to I2E promoter of MnSOD of PC3 cells. (**H**) Representative Western blots and quantitative analysis of protein expression of PCa cells. *n* = 3. * *p*-value ≤ 0.05 when compared with vehicle.

To determine whether BET exerts a different effect on RelB expression, we measured the level and activity of RelB. As shown in Figure 4, RT-PCR analysis demonstrated that BET and RT + BET treatments resulted in an increase in RELB mRNA in non-cancer cells but a decrease in PCa cells compared to vehicle treatment (Figure 4A,E). BET + RT treatment in the NF-kB binding assay supported that BET + RT treatment increased RelB-DNA binding activity in PrEC and PZ cells but not in LNCaP, DU145, or PC3 cells (Figure 4B,F). The combination of BET and RT further promoted RelB-DNA activity in non-PCa cells while it suppressed RelB-DNA activity in PCa cells, in comparison to BET or RT alone. To test whether BET + RT treatment affects RelB downstream targets, PC3 cells and PZ cells were subjected to further studies. Chromatin immunoprecipitation (ChIP assay) with the RelB antibody to the promoter/enhancer (I2E) of the *SOD2* gene was quantified by qPCR. As expected, the RelB-transcriptionally regulated *SOD2* gene significantly increased in PZ cells in response to BET treatment (Figure 4C), although it was not statistically significant, but the SOD2 qPCR was slightly reduced in PC3 cells, compared to vehicle (Figure 4G). Western blots confirmed an increase in RelB and MnSOD protein levels in non-cancer cells, PZ and PrEC (Figure 4D), and a decrease in RelB and MnSOD protein levels in PC3 cells with BET ± RT treatments (Figure 4H). In addition to β-actin, GAPDH was also utilized as an internal control in PC3 cells (Supplementary Figure S5). Overall, these data suggest that BET combined with RT affects RelB at the mRNA level and protein level, with a consequent increase in MnSOD protein expression. These results suggest that RelB plays a role as a mediator for the difference in responses of normal prostate cells and PCa cells to BET, especially when combined with RT.

### 2.4. RNA Sequencing Analysis Identifies BLNK as a Novel RelB Partner

**Figure 5:**
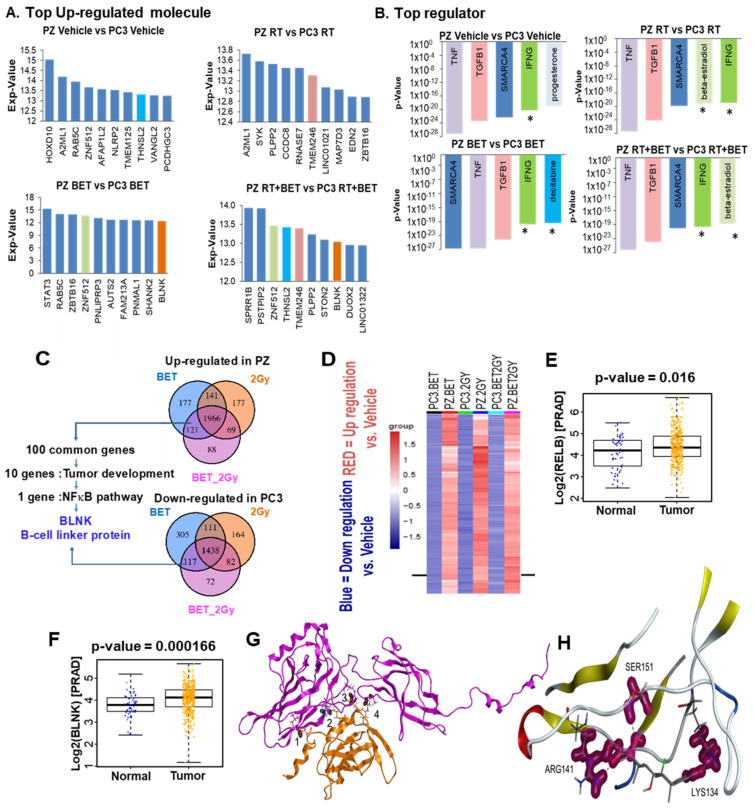
RNA sequencing identifies BLNK as a novel complementary protein that is upregulated by BET-mediated RelB expression. (**A**) Upregulated molecules that were most significantly changed (threshold exp-value 10). The rank order is determined by the indicated *p*-values. (**B**) 5 regulator pathways that were most significantly changed (threshold *p*-value 1.3). All these top regulators contain NF-kB family, and regulator star (*) contains BLNK. (**C**) Venn diagrams identified BLNK as the one of the proteins that is upregulated in PZ cells and downregulated in PC3 cells. (**D**) Heat map demonstrated ~2000 common genes that are diversely expressed in PC3 and PZ cells by all the treatments. Red = Upregulation vs. vehicle; Blue = Downregulation vs. vehicle, in PZ vs. PC3 cells. (**E**) RelB and (**F**) BLNK in PCa (*n* = 499) vs. normal prostate (*n* = 52) from TCGA database vs. vehicle. PARD = Prostate Adenocarcinoma. RELB and BLNK expressions are presented in log2-transformed transcripts per kilobase million (TPM) values. *p*-value calculated from linear mixed model. (**G**) PPI between RELB (magenta) and BLNK (orange). Contacts (1–4) given following: (1) Hydrogen bond at Cys 343 (BLNK) and Leu127 (RelB); (2) Ionic bond at Arg427 (BLNK) and Glu290 (RelB); (3) Hydrogen bond at His431 (BLNK) and Arg136 (RelB); and (4) Hydrogen bond at Arg448 (BLNK) and Cys389 (RelB). (**H**) Docking and binding analysis with ZDOCK and PyMol suggesting the interaction at the Y300 residue of RelB and the conserved R32 and R51 residues of BLNK.

While it is well-established that RelB regulates MnSOD [16], little is known about the RelB partner that causes different responses in non-cancer cells versus PCa cells. We treated PC3 and PZ cells with BET (5 µM) ± RT (2 Gy) for 24 h, performed RNA sequencing, and conducted differential expression testing and pathway enrichment analysis (Supplementary Figure S6) to identify the RelB partner. Using this unbiased approach, we identified the top upregulated molecules that were significantly changed in PZ cells compared to PC3 cells. BLNK was the mRNA that significantly increased, particularly with BET treatment, both with and without RT (Figure 5A). In addition, several inflammatory response mediators were also key upstream regulators that were differentially modulated in PZ and PC3 cells. The five top regulators based on the order of *p*-value are shown in Figure 5B. This finding is consistent with the role of RelB in immune cells. Next, we identified ~100 transcripts in which expression commonly changes in PC3 and PZ cells after BET ± RT, as shown in the Venn diagrams (Figure 5C). These common transcripts include positive and negative transcriptional regulators that may participate in response to BET ± RT. The inverse expression of these genes in PZ and PC3 cells suggests the possibility that they have roles as RelB partners that cause the different responses of PZ and PC3 cells to BET ± RT. Among these common genes, we identified an adaptor protein of the antigen receptor of B-cells, BLNK, as the leading protein whose expression pattern was changed by BET and is associated with the NF-κB pathway. As illustrated by a heat map (Figure 5D), BLNK expression level decreased in PC3 cells while it increased in PZ cells with BET and BET + RT treatment, similar to RelB. The TCGA database shows that RelB and BLNK expressions are higher in PCa patients than in normal individuals (Figure 5E,F). These results suggest that BLNK could be a novel complementary partner of RelB upon BET treatment.

### 2.5. Protein–Protein Interaction (PPI) Prediction between RELB and BLNK and Binding of BET to RelB

We further demonstrated the interaction between RELB and BLNK through text mining with two different tools: (1) STRING database and (2) precomputed interaction predictions from SPRINT. For score relevance, the following metrics were collected. The average, high, and low scores were collected for all RELB predictions, all BLNK predictions, and all predictions in the entire dataset and reported in Supplementary Table S1. The physical interaction partners for each were collected from STRING, while the SPRINT scores were collected for each of the pairs. (Supplementary Tables S2 and S3). A summary of the number of times two residues were predicted to form a contact using the top five predicted models from each tool is given in Supplementary Table S4. Many of the contacts that formed out of multiple modeling attempts appear in the Molecular Operating Environment (MOE, Chemical Computing Group Inc., Montreal, QC, Canada) top model. With the highest interaction score of 72.4458, a figure of the top model from MOE is demonstrated in Figure 5G, and the contacts are: (1) Hydrogen bond at Cys 343 (BLNK) and Leu127 (RelB); (2) Ionic bond at Arg427 (BLNK) and Glu290 (RelB); (3) Hydrogen bond at His431 (BLNK) and Arg136 (RelB); and (4) Hydrogen bond at Arg448 (BLNK) and Cys389 (RelB). In the virtual high-throughput screen, BET ranks the best at 7.91%, with only one of the inactive compounds ranking better (Supplementary Table S5). Figure 5H demonstrates a potential hydrogen bond docking of BET to RelB on Arg141, Ser151, and Lys134.

### 2.6. Interaction of Nuclear RelB and BLNK Confers Differential Responses to BET

**Figure 6:**
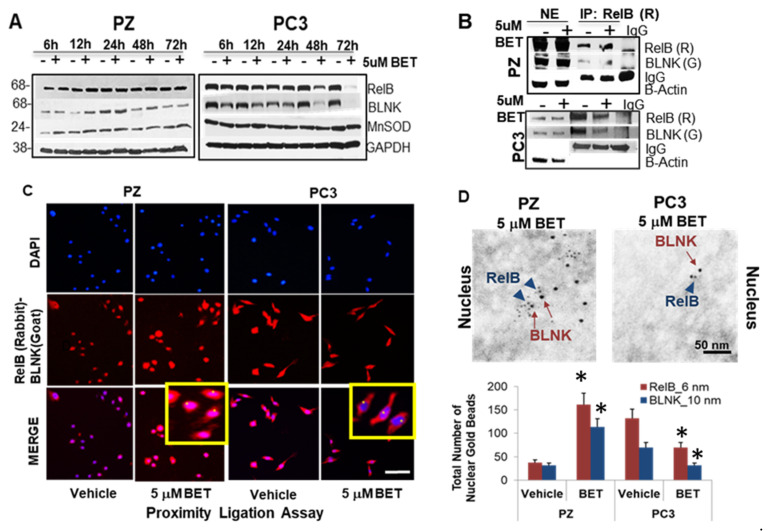
RelB and BLNK exert their roles reversely in non-PCa cells vs. PCa cells with BET treatment. Cells were treated with BET for 24 h and then harvested for analysis. (**A**) Western blots of BLNK, RelB, and MnSOD upon BET treatment. (**B**) IP with RelB antibody in whole cells nuclear fraction (NE) after treatment with BET. R = Anti-rabbit antibody. G = Anti-goat antibody. (**C**) Proximity Ligation assay. Red = Binding of RelB and BLNK. Blue = Nucleus. Inserts indicate the binding of RelB and BLNK in the nuclei of PZ cells (yellow) but not in the nuclei of PC3 cells (yellow). Bar = 50 μm. (**D**) Representation of double immunogold electron microscopy of cells and quantification of gold bead number in nuclei. PZ cells or PC3 cells were labeled with anti-rabbit RelB antibody (6 nm gold beads, arrow heads) and anti-goat BLNK antibody (10 nm gold beads, arrow). The gold beads were localized primarily in the nuclei of PZ cells after BET treatment but less in the nuclei of PC3 cells. * *p*-value ≤ 0.05 when compared with vehicle. Twenty nuclei were counted per group.

To confirm the complementary relationship between RelB and BLNK, Western blot analysis and immunoprecipitation (IP) were employed. The protein levels of RelB, BLNK, and MnSOD increased in PZ cells (up to 48 h) but decreased in PC3 cells (up to 72 h) with BET treatment (Figure 6A). An IP analysis of the RelB antibody revealed that BLNK interacts with RelB in PZ and PC3 cells (Figure 6B). Treatment with BET resulted in an increase in the RelB-BLNK interaction in normal cells but a decrease in cancer cells, particularly in the nuclear fraction. Utilizing the Duolink^®^ Proximity Ligation Assay (PLA), as indicated by red fluorescence expression, the nuclear interaction of RelB and BLNK was enhanced in PZ cells but suppressed in PC3 cells when treated with BET (Figure 6C). PZ and PC3 cells stained with either RelB or BLNK antibody alone did not show significant red fluorescence staining (Supplementary Figure S7).

Due to their labeling sensitivity and high spatial resolution [22], gold particles of different sizes, conjugated to different species of primary antibodies, were used to distinguish labeling of RelB (6 nm gold beads) and BLNK (10 nm gold beads) (Figure 6D). Consistently, RelB and BLNK were found in the nuclei and the cytoplasm of PZ cells after BET treatment; in contrast, less RelB and BLNK were found in the nuclei and the cytoplasm of PC3 cells with BET treatment. Normal rabbit serum or normal goat serum did not show significant gold bead labeling (Supplementary Figure S8). A quantification of gold beads confirmed that RelB and BLNK protein levels were significantly increased in PZ cells’ nuclei and significantly decreased in PC3 cells’ nuclei (Figure 6D). Hence, the RelB-BLNK level in the nuclear compartment was altered as part of the response to BET.

### 2.7. RelB-BLNK Axis Acts as a Central Regulator in Cellular Responses to BET Treatment

**Figure 7:**
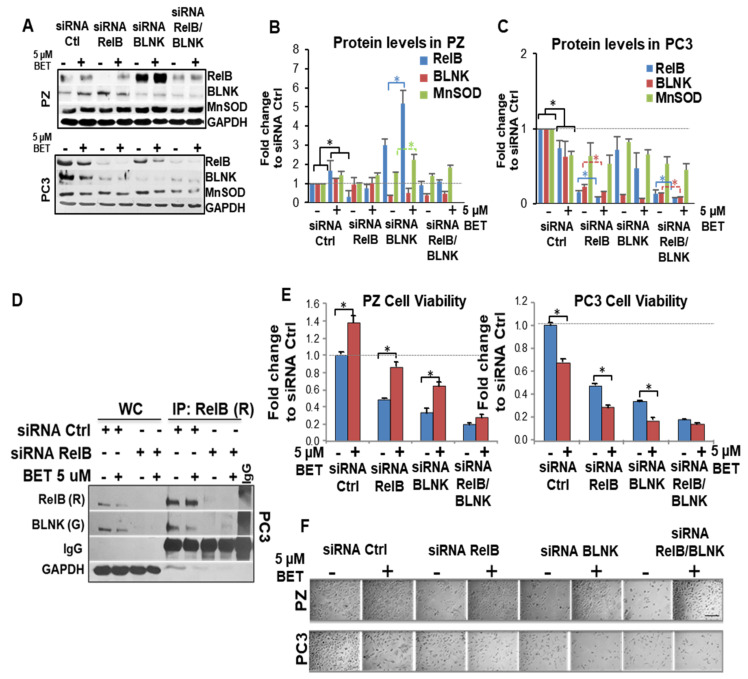
BLNK interaction with RelB is a signal for BET protection of RT-induced injury in normal tissues. Cells were transfected with siRNA (Santa Cruz biotechnology) for 48 h and then treated with BET for 24 h. Western blot analysis (**A**) and protein quantification of (**B**) PC3 and (**C**) PZ cells. *n* = 2. (**D**) IP analysis confirmed a decrease in RelB:BLNK axis with RelB knockdown. WC = Whole cell lysates. R = Anti-rabbit antibody. G = Anti-goat antibody. (**E**) Trypan Blue assay and (**F**) representative photograph indicates a decrease in cell viability with siRNA against RelB, BLNK, or RelB + BLNK with or without BET treatment. *n* = 3. * *p*-value < 0.05 vs. non-BET. Scale bar = 50 µm.

To gain insight into the role of the RelB-BLNK axis on cell viability, RelB and/or BLNK were knocked down in PZ and PC3 cells with siRNAs, from Santa Cruz Biotechnology (Dallas, TX, USA) (pool of three siRNA duplexes) and Thermo Fisher (Waltham, MA, USA) (a mixture of three individual siRNAs). Western blots and quantification demonstrated an approximately 70–80% decrease in RelB and BLNK expression in both PZ cells and PC3 cells (Figure 7A–C and Supplementary Figure S9). Negative control (siRNA scrambled) did not change the expression level of RelB or BLNK (data not shown). Interestingly, BET-mediated changes in RelB and BLNK were independent of one another. BET induced RelB expression in PZ cells, even with the BLNK knockdown. Although it was not statistically significant, BET suppressed RelB expression in PC3 cells, even with the BLNK knockdown, and significantly suppressed BLNK expression, even with the RelB knockdown. An IP analysis showed a decrease in RelB-BLNK interaction with RelB knockdown (Figure 7D). Further, a trypan blue assay (Figure 7E) and bright field contrast images (Figure 7F) indicated that the level of BET-mediated cytotoxicity in PC3 cells was decreased, while the level of BET-increased cell viability in PZ cells was decreased by RelB knockdown or BLNK knockdown. The simultaneous knockdown of RelB and BLNK prevented BET-induced PZ cell viability and BET-suppressed PC3 cell viability. Thus, RelB and BLNK collaborate to regulate the cell viability of PZ cells and PC3 cells during BET treatment.

## 3. Discussion

In this study, we screened a library of FDA-approved drugs and found BET exhibiting the following properties: (1) It enhances cancer cell death by radiation and protects non-cancer cells against radiation toxicity; (2) It increases H_2_O_2_ production in both cancer cells and non-cancer cells. These properties support our hypothesis that the selective sensitization of cancer cells to ROS-generating therapeutics may drive cancer cells, which already have high constitutive oxidative stress levels, toward death. Normal cells, however, are able to maintain redox homeostasis through adaptive responses.

BET is a glucocorticoid that is approved for treatment of inflammation and cancer of the hematopoietic system [23,24]. Members of the glucocorticoid family, such as prednisone and dexamethasone, have been shown to lessen pain in hormone-refractory PCa patients [25,26]. Our study is the first to demonstrate that BET protects normal prostate cells from RT-induced injury but exacerbates the susceptibility of PCa cells to RT-induced death. Theoretically, BET could mediate H_2_O_2_ production when the steroids bind to a plasma membrane [27,28]. Due to the unique structure of steroids [29], BET-mediated H_2_O_2_ production could be due to the redox cycle of BET, prooxidants/antioxidants that can elicit a protective response from normal cells but add injury to tumor cells after RT. This key concept of using redox-cycling compounds, in addition to BET, as adjuvant therapy for targeting cancers are underway in our laboratory and others. Importantly, our studies show that upon RT, BET-mediated H_2_O_2_ production triggers RelB-mediated MnSOD protein expression in non-cancer cells but not in PCa cells. Please note, the change in MnSOD protein expression upon BET + RT is greater than SOD2 mRNA, and the change in MnSOD protein expression is significantly decreased in PC3 cells but slightly decreased in LNCaP cells and DU145 cells. The differences in the degree of MnSOD protein expression could be due to the differences in the redox state of each cell type, their genetical background, and other transcription factors of MnSOD, such as SP-1. Hence, upregulation of MnSOD, a primary mitochondrial antioxidant, potentially protects against RT-induced mitochondrial damage and non-cancer cell death in response to RT. It is noteworthy that, regardless of the basal cellular redox state, BET activates similar H_2_O_2_ production in both PZ cells and PC3 cells. These data suggest the possibility that BET-mediated ROS production initiates the protective response of MnSOD in PZ cells, and the increases in MnSOD-mediated H_2_O_2_ contribute to the killing effect in cancer cells. Interestingly, RT generates ROS, and RT also induces RelB in both normal and cancer cells. It is possible that the observed differential effects of BET and RT on RelB expression in PCa cells are caused by the different mechanisms of how BET produces ROS versus RT.

The vertebrate Rel/NF-kB transcription factors (c-rel, RelA, RelB, NF-kB1 (p50/p105) and NF-kB2 (p52/p100)) play vital roles in immune, inflammatory/stress responses, and are key to tumor survival [30,31]. RelB transcription activity is dependent on a TATA-less promoter containing two NF-kB binding sites. One NF-kB site primarily binds p50 while the other binds to and is transactivated by RelB [32]. Structurally, RelB requires both its N-terminal and C-terminal for transcriptional activation [33]. RelB also contains a leucine zipper at its N-terminal. Due to its unusual structure, RelB only dimerizes with p50 or p52 and cannot form homodimers [34,35]. We have previously reported that RelB regulates MnSOD [16]. Here, we demonstrate that BLNK serves as a RelB complementary partner that regulates MnSOD expression during BET + RT treatment. Based on a PPI model, we propose that BLNK interacts with RelB at the protein level and co-modulates the expression of the *SOD2* gene via the RelB binding site at the promoter/enhancer of *SOD* during BET + RT. The simultaneous knockdown of both RelB and BLNK upon BET treatment alone, although not statistically different, slightly promotes MnSOD protein expression in PZ cells and slightly suppresses MnSOD protein expression in PC3 cells (Figure 7B,C); we propose that the other transcription factors that regulate MnSOD or unidentified RelB partners could compensate for the slight changes observed in MnSOD. Since we observed the inhibition of (1) BET-induced cell death in PC3 cells and (2) the BET-promoted cell viability in PZ cells, with the slight change in MnSOD, we propose that other unidentified RelB regulators/partners or RelB downstream targets could also contribute to the inhibiting effect of BET. For example, RelA is a RelB regulator; however, we did not observe consistent changes in RelA activity in non-PCa cells or PC3 cells (Supplementary Figure S10). Thus, the changes in RelB expression by BET may be RelA-independent. Please note, in addition to RelB and BLNK, the observed phenotype changes upon BET and BET + RT could be caused by other proteins and pathways since RNA sequencing show at least 100 genes that increase in PZ but decrease in PC3 upon these treatments (Figure 5A–C). Future studies focusing on other pathways, such as TNF-alpha, would add to the knowledge of additional mediators responsive to redox-cycle agents. These possibilities are intriguing and would be an interesting topic for future studies.

BLNK, an adaptor protein of the antigen receptor on B-cells, participates in the PI3K/Akt pathway that leads to the activation of JNK and ROS generation, and promotes B-cell proliferation/survival [36]. The knockdown of BLNK causes apoptosis in B-cell leukemia [37]. One mechanism of BLNK that has not been previously explored is its partnership with RelB. Notably, with the use of RNA sequencing, coupled with extensive verification by PLA, double immunogold labeling, and IP, we propose that BLNK is a novel complementary partner of RelB, especially upon BET/RT-mediated H_2_O_2_ production. Since the ChIP assay with the BLNK antibody did not yield a DNA fragment of the *SOD2* promoter/enhancer region (data not shown), it is likely that BLNK does not directly bind with the *SOD2* gene, but only binds with RelB as a complementary partner during BET treatment. These findings raise the possibility that BLNK is involved in sensing and modulating cellular redox status, which enables cells to adapt their reliance on ROS signaling for pro-proliferative and anti-apoptotic activities. In addition, BLNK could promote RelB translocation to the nucleus since an increasing BLNK level correlated with nuclear RelB.

**Figure 8:**
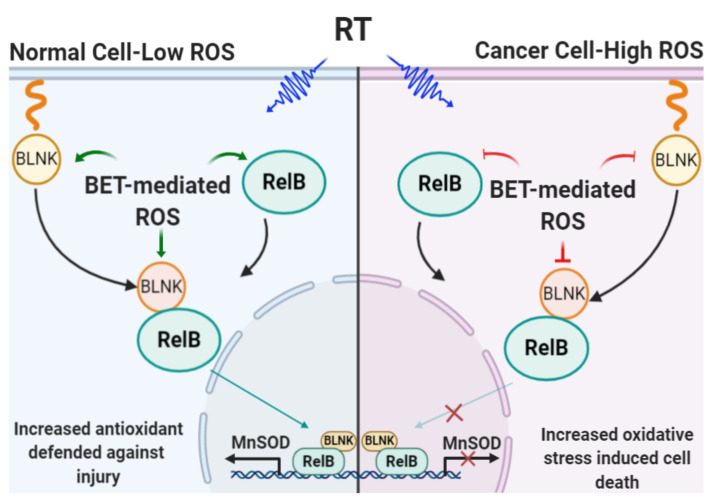
Schematic of how BET-mediated ROS production sensitizes PCa cells toward death caused by RT while protecting non-PCa cells against injury from RT off-target effects. Aberrant redox homeostasis of cancer cells enables redox-modifying agent BET to enhance radiation therapy efficacy by selective sensitization. In normal cells under physiologic conditions, cellular redox status is kept at a low oxidizing level. A shift in cell redox status toward an oxidizing condition, from BET + RT, will stimulate the expression of RelB-BLNK and translocation to the nucleus, which leads to upregulation of the antioxidant system, including MnSOD. The upregulation of MnSOD maintains redox status in normal cells and promotes cell survival. To the contrary, cancer cells are usually under high oxidizing conditions. A comparable shift in ROS levels modulated by BET + RT to an extreme oxidizing condition will cause cell death. Green arrows = activation; Red line = inhibition.

ROS production is a previously unrecognized property of BET. The proposed ROS- mediated mechanisms showing how BET protects normal cells from RT-induced injury while exacerbating RT-induced cancer killing are illustrated in Figure 8. BET-mediated ROS production enhances the RelB-BLNK interaction in normal prostate cells but suppresses RelB-BLNK in PCa cells. Our results support the critical concept that the intrinsic differences in cellular redox status, the RelB-BLNK axis, and the different adaptive responses to prooxidants by normal cells and cancer cells can be used to identify drugs that would protect against normal tissue injury while increasing RT efficacy. The efficiency of RT in controlling localized PCa could very likely contribute to a large number of PCa survivors enjoying long, productive lives after cancer therapy. Thus, redox-active agents that protect normal tissue while not reducing the effect of RT are urgently needed. Given that BET regulates RelB-BLNK complex levels and considering the high expression levels of RelB and BLNK in PCa patients, as determined by the TCGA database, this study highlights a novel precision medicine approach to reduce the side effects of RT in PCa patients.

## 4. Materials and Methods

### 4.1. Cell Culture and Reagents

Human PCa cell lines, PC3 cells, DU145 cells, LNCaP-derived cell line, LNCaP-C4-2B (C4-2B), LNCaP cells, and PZ cells were obtained from American Type Culture Collection (ATCC). These cell lines were authenticated using the Short Tandem Repeat (STR) profiling service from ATCC and were routinely checked for mycoplasma contamination (MycoSensor PCR assay, Thermo Fisher, Florence, KY, USA). Cells were cultured in RPMI 1640 medium as previously described [9]. Cells passaged fewer than 15 times were used in all experiments. PrECs were obtained from Lonza (Basel, Switzerland) and maintained in the manufacturer’s suggested media. Chemicals and reagents were purchased from Sigma Chemical Corp (St. Louis, MO, USA). Cell culture reagents were purchased from Thermo Fisher Scientific. Primary and secondary antibodies were obtained from Santa Cruz Biotechnology unless otherwise specified. Gold conjugated secondary antibodies were obtained from BB International Cardiss, United Kingdom. Betamethasone was obtained from U.S. Pharmacopeia (Rockville, MD, USA) for in vitro experiments (>99% purity, powder, USP/FCC grade) and from Cardinal Health (Dublin, OH, USA) for animal experiments (injectable, Betamethasone sodium phosphate). BET was freshly prepared prior to application.

### 4.2. Cell Survival Analysis and Radiation Treatment

Depending on the cell type, cell survival fractions were quantified by colony survival (6-well plates, 100 to 500 cells per well) or MTT assay (96-well plates, 1000–5000 cells per well) (Trevigen, Gaithersburg, MD, USA) [38]. Cells were pretreated with 200 units PEG-CAT for 24 h prior to BET treatment. The surviving fraction (SF) was calculated as the ratio of the number of colonies formed to the number of cells effectively plated, with a correction for the plating efficiency (PE) as follows: SF = colonies counted/cells seeded × (PE/100) [39]. For combination therapy, RT was performed at the same time as BET treatment by a 250 kV X-ray machine (Faxitron X-ray Corp, Tucson, AZ, USA), with peak energy of 120 kV, 0.05 mm Al filter, at a dose of 0 to 6 Gy. D0 values (the dose to reduce survival fraction to 37%) were calculated from the linear portion of Linear–Quadratic Survival Curves [21]. The linear–quadratic (LQ) model utilizes terms α (single-hit kill) and β (two-hit kill), which correlate with low-dose killing and high-dose killing, respectively. The LQ model can be used to determine biologically equivalent doses between various dose fractionation schemes.

### 4.3. Quantification of H_2_O_2_ Levels

Cells were incubated with 50 μM Amplex Red reagent (Thermo Fisher) at 37 °C for 30 min. Fluorescence was detected at ex/em 550/590 nm using a Gemini XPS Microplate Reader (Molecular Devices, San Jose, CA, USA). To account for H_2_O_2_-specific fluorescence, PEG-CAT was used as described above. Intracellular production of H_2_O_2_ was estimated using a sensitive assay based on the aminotriazole-mediated inactivation of CAT, as previously described by Wagner et al. [40]. Cells at 70–90% confluence in 100 mm tissue culture dish were treated with BET for 6 h, and 20 mM 3-aminotriazole (3-AT) was then added and incubated at 37 °C for 0, 5, 10, 15, 30, and 45 min. Cells were rinsed twice and harvested with ice-cold 50 mM phosphate-buffered saline (PBS), and pH 7.4 and pellets were collected. H_2_O_2_ concentration was calculated by kinetic analysis of the rate of decrease in CAT activity [41]. Spectrophotometric CAT activities were initiated by addition of 30 mM H_2_O_2_ and the loss of absorbance at 240 nm at 25 °C was monitored on a Beckman DU-600 UV–Vis spectrophotometer (Beckman–Coulter, Fullerton, CA, USA). Initial CAT activities were calculated by fitting experimental data to the first-order kinetics and expressed as catalase k mU/mg cell protein.

### 4.4. Screening Assay of FDA-Approved Drugs

Enzo Screen-Well^®^ FDA-approved Drug Library Version 2 (786 approved drugs, Supplementary Excel File) was obtained from the Center for Pharmaceutical Research and Innovation, University of Kentucky, Lexington, KY. Cells were plated (2000 cells/well, 96-well plates in duplicate) and incubated with drugs in dimethyl sulfoxide (DMSO) (screening dose at 10 µM). MTT and Amplex red assays were performed after 24 h incubation. In addition, RT (4 Gy) in combination with the selected compounds was applied to PZ cells (1000 cells/well) on a separate set of plates. MTT assay was then performed 96 h after RT. Top candidate drugs were selected and validated with different concentrations varying from nm to µM.

### 4.5. Western Blots and IP

Western blot and IP protocols have been previously described [42]. Protein levels were determined by Bradford assay. Primary antibodies against RelA, RelB (Thermo Fisher), BLNK (Abcam, Cambridge, UK), MnSOD (Millipore Sigma, Darmstadt, Germany), and GAPDH or β-actin (Sigma) were used. Western blots were visualized using an enhanced chemiluminescence detection system (Amersham Pharmacia Biotech, Amersham, UK). For the IP experiment, a nitrocellulose membrane was first incubated with RelB antibody, striped, and re-probed with BLNK antibody (due to close MW of RelB and BLNK).

### 4.6. Real-Time PCR

mRNA was isolated from cells using a MagNA Pure Compact RNA Isolation Kit (Roche, Basel, Switzerland), reverse-transcribed using a TaqMan reverse transcription kit (Thermo Fisher), and analyzed using a LightCycler^®^ 480 Real-Time PCR System (Roche) with gene-specific primers (Invitrogen, Carlsbad, CA, USA) as follows:

RELB: 5′-cacttcctgcccaaccac-3′ (forward) and 5′-gacacggtgccagagaaga-3′ (reverse)
BLNK: 5′-cgagtgctcatctggattttcc-3′ (forward) and 5′-agagagccctgctgacga-3′ (reverse)
RELA: 5′-cgggatggcttctatgagg-3′ (forward) and 5′-ctccaggtcccgcttctt-3′ (reverse)
SOD2: 5′-agcatgttgagccgggcagt-3′ (forward) and 5′-aggttgttcacgtaggccgc-3′ (reverse)
β-actin: 5′-ccaaccgcgagaagatga-3′ (forward) and 5′-ccagaggcgtacagggatag-3′ (reverse)
The relative mRNA copy number was analyzed by the 2-^ΔΔ^Ct method [43].

### 4.7. NF-kB (RelB-DNA) Binding Assay and ChIP

Nuclear extracts were prepared and RelB-DNA binding activities measured using an ELISA-based TransAM NF-kB Family kit (Active Motif) [9]. A Pierce^TM^ Agarose ChIP Kit (Thermo Fisher) was used to study RelB-mediated transcriptional regulation. Chromatin that was pulled down using a RelB antibody and a DNA fragment containing an NF-kB element located on the I2E promoter/enhancer region of the human *SOD2* gene was analyzed by quantitative PCR. PCR primer sequences were 5′-cggggttatgaaatttgttgagta-3′ (upper strand) and 5′-ccacaagtaaaggactgaaattaa-3′ (lower strand). MnSOD exon 2 was amplified as an untargeted control. Primer sequences were 5′-tgaccgggctgtgctttctcg-3′ (upper strand) and 5′-actgcctcccgccgctcagcc-3′ (lower strand). ChIP-qPCR data were normalized by input preparation.

### 4.8. RNA Sequencing and Data Analysis

mRNA was isolated using a MagNA Pure Compact RNA Isolation Kit. RNA concentration and RNA integrity (RIN) were confirmed using Bioanalyzer (Agilent). Only samples with RIN > 8.5 were used for Whole Transcriptome RNA Sequencing. Paired-end sequencing strategy was performed with read length 100 bp, using entire FlowCell HiSeq 2500 Rapid Run (Illumina Inc. (San Diego, CA, USA)). Library preparations (TruSeq Standard Total RNA), RNA sequencing, and quality control were performed by the Oncogenomics Shared Resource Facility. For each library, at least 40 million reads were obtained. Raw sequencing data were transferred to the Biostatistics and Bioinformatics facility for data processing and analysis. Sequencing reads were trimmed and filtered using Cutadapt (v1.4) [44]. Reference transcripts were extracted using the Ensembl GRCh38 annotation gtf file. Transcriptome alignment was carried out using STAR [45], and gene abundance was estimated using RSEM [46]. We performed differential expression analysis using edgeR [47] to compare the two cell lines (PC3 cells and PZ cells) under each treatment condition. Significantly up- or downregulated genes were determined as fold change ≥ 2 and false discovery rate q-value < 0.05.

### 4.9. Proximity Ligation Assay 

Cells (1 × 10^3^) were plated on slide wells, permeabilized, and blocked 24 h after treatment. Duolink In Situ kit was employed (Sigma-Aldrich, St. Louis, MO, USA). Secondary antibodies coupled with oligonucleotides (PLA probe) were applied to anti-rabbit RelB and anti-goat BLNK binders to reveal sites of RelB and BLNK co-localization. PLA signals were detected by Olympus IX71 fluorescence microscope as red fluorescence.

### 4.10. Double Immunogold Labeling of RelB and BLNK

Cells (5 × 10^5^) were plated, treated with BET for 24 h, fixed in Carson Milonig fixative, dehydrated in graded ethanol, embedded in LR white resin, and mounted on 1% collodion membrane–coated nickel grids [48]. Pre-incubations of anti-rabbit RelB antibody with ultra-small 6 nm gold conjugated F(ab′)2 fragments of anti-rabbit antibody, or anti-goat BLNK antibody with 10 nm gold conjugated F(ab′)2 fragments of anti-goat antibody, were performed at room temperature for 3 h. Grids were then incubated overnight with RelB/BLNK-gold conjugated antibody complexes at 4 °C, washed, and counterstained with 2% uranyl acetate. Grids were photographed with a Hitachi H-600 electron microscope (Schaumburg, IL, USA; amplification 60,000X). For quantitative comparison, 20 cells from each treatment group were analyzed.

### 4.11. Morphometric Quantification by EM

Prostate tissues were fixed, embedded, and processed for EM [48] and observed with a Hitachi H-600 electron microscope. At least 300 cells were quantified. Mitochondria with any of the following criteria were used for mitochondrial damage: mitochondrial swelling, mitochondria with loss of cristae, degeneration of mitochondria, lysosomal degradation of mitochondria, vacuolization in mitochondria. Cytoplasm with any of the following criteria were used for cytoplasmic damage: intracytoplasmic vacuolization, intracellular edema, disruption of cell membranes. Cells with at least 50% mitochondrial and/or cytoplasmic damage were considered damaged cells. Damaged cells were counted and reported as % of damaged cells.

### 4.12. Animal Studies and Treatment Regimens

All animal experimental procedures were approved by the Institutional Animal Care and Use Committee of the University of Kentucky (Lexington, KY, USA), approval Protocol No. 01077M2006. For non-tumor experiments, four- to five-week-old male NCRNU (nu/nu athymic nude) mice were obtained from Taconic (Hudson). Mice were intraperitoneally injected (i.p.) daily for 5 days as follows: (1) vehicle (saline, *n* = 3); (2) BET (20 mg/kg, *n* = 5); (3) vehicle and fractioned 2 Gy RT (total of 10 Gy, *n* = 5); (4) BET (20 mg/kg) and fractioned 2 Gy RT (total of 10 Gy) (*n* = 5). The Varian TrueBeam linear accelerator system (Varian Medical System Inc., Palo Alto, CA, USA, 6-MeV electron beam) was used for locally irradiated prostate organ in non-tumor mice, and the dosimetry was calculated by a physicist, Dr. Wei Lou. Briefly, four mice were placed in a radiation jig and covered by 15 mm of Cerrobend shielding. The prostate was positioned beneath each 2.5 cm diameter aperture in the Cerrobend shielding, thus allowing the prostate to be irradiated while shielding the remainder of the animal. For combination treatments, RT was performed 1 h after BET injection. The minimum dose that did not cause toxicity was used as the BET dose. Prostate tissues and serum were harvested one week after treatment was completed.

For tumor-bearing mice, ~2 × 10^6^ PC3 cells mixed in Matrigel (BD Biosciences, San Jose, CA, USA) (50 µL:50 µL) were subcutaneously injected into the right flank. Tumor volumes were routinely measured and calculated (length × width × width × 0.52). Animals with an average tumor size of 200 mm^3^ were randomized into 4 groups (*n* = 10) and treated as indicated above. The s.c. tumors were positioned beneath each 2.5 cm diameter aperture in the Cerrobend shielding. After treatment, the mice were observed daily until the tumor reached the maximum size of 1500 mm^3^, at which time they were humanely killed. The tumor tissues were collected for protein analysis.

### 4.13. Measurement of 4HNE-Adducted Proteins

Mouse blood was collected one week after the treatment. Serum 4HNE levels were measured using the immunoblot technique previously mentioned [49].

### 4.14. siRNA Transfection and Cell Viability by Trypan Blue

siRNA RelB, siRNA BLNK, and scrambles were purchased from Santa Cruz Biotechnology and Thermo Fisher. Santa Cruz Biotechnology siRNA is a pool of three siRNA duplexes as indicated below (all sequences are provided in 5′ → 3′ orientation):

RelB siRNA (sc-36402) is a pool of three different siRNA duplexes:
A. Sense: GCAACAUGUUCCCCAAUCATT. Antisense: UGAUUGGGGAACAUGUUGCTT.
B. Sense: CGUGCACUAGCUUGUUACATT. Antisense: UGUAACAAGCUAGUGCACGTT.
C. Sense: CUCCAGUAGGAUUCGGAAATT. Antisense: UUUCCGAAUCCUACUGGAGTT.

BLNK siRNA (sc-29810) is a pool of three different siRNA duplexes:
A. Sense: CGUGACCACUGGACAGUUATT. Antisense: UAACUGUCCAGUGGUCACGTT.
B. Sense: GCAAGACACUUCCCAGUAATT. Antisense: UUACUGGGAAGUGUCUUGCTT.
C. Sense: AGUUGCCUCUCAACAGAAUTT. Antisense: AUUCUGUUGAGAGGCAACUTT.

In contrast, Thermo Fisher siRNA is a mixture of three individual pre-design *Silence* Select siRNAs (RelB: ID11917 + ID11918 + ID11919, BLNK: IDs26556 + IDs26557 + IDs26558) (Supplementary Figure S9). Cells were seeded at 6 × 10^4^ cells/well in 6-well plates and were transfected with 50 µM/mL siRNA(s), using siRNA transfection reagent (Santa Cruz), for 48 h. Fresh media with BET were added 24 h before using Trypan Blue Solution (0.4%) to assess viable cells by hemocytometer [50].

### 4.15. Protein–Protein Interaction Prediction between RELB and BLNK

Two different tools were used to look for evidence of a protein–protein interaction (PPI) between RELB and BLNK. One was the STRING database (string-db.org) [51,52,53]. STRING combines data from multiple sources and gives a combined score of interactions based on multiple types of inferences, coming from experimental findings, text mining, and databases. The other was precomputed interaction predictions from SPRINT (Scoring PRotein INTeractions) [54]. SPRINT predicts PPIs on a genome-wide scale using sequence alone. It uses machine learning, known interacting proteins, and common sequences to make predictions. An analysis of produced scores and scores of known interactions was performed. A higher score correlates with a high potential of PPI. To model the potential PPI contacts between RELB and BLNK, the structured region of the AlphaFold models of both proteins was used. Protein–protein docking was performed in both Molecular Operating Environment (MOE) and the ZDOCK Server (http://zdock.umassmed.edu (accessed on 9 September 2021)) [55]. The contacts between RELB and BLNK were then calculated and visualized using MOE.

### 4.16. Binding of Betamethasone to RELB

A docking analysis was performed to assess the likelihood of BET binding to RELB. The 3-dimensional structure of BET was obtained from DrugBank (Accession Number DB00443). In order to create a ranking and comparison, five inactive compounds and one active compound to RELB were identified from PubChem Bioassay (PubChem AID 1614608) [56], and a background of approved drugs was obtained from BindingDB (https://www.bindingdb.org/bind/ByFDAdrugs.jsp (accessed on 24 August 2021)) [57]. To perform the docking analysis, the structured region (higher model confidence) of the alpha-fold [58] structure of RELB was used. MOE [59] was used to determine putative binding sites, and VinaMPI [60,61] was used to score the binding potential.

### 4.17. Statistical Data Analyses

At least three independent experiments were conducted for each in vitro study. ANOVA and Tukey’s HSD tests were used to compare the mean of log-transformed tumor size at each time point across multiple treatment groups. Kaplan–Meier curves and logrank tests were used to compare the times to tumor size reaching 1500 mm^3^. TCGA prostate adenocarcinoma RNAseq data of 499 tumors (from 495 patients) and 52 matched normal samples were downloaded from the Genomic Data Commons (https://gdc.cancer.gov (accessed on 14 December 2019). RELB and BLNK expressions are presented in log2-transformed transcripts per kilobase million (TPM) values. The *p*-value is calculated from the linear mixed model. The linear mixed model was used to compare RELB and BLNK expressions of tumor and normal samples. CFAssay for R (R) (20) was used for the statistical analysis of the colony formation assay. Statistical analyses were performed with SPSS 14 software (SPSS, Inc., Chicago, IL, USA), SAS 9.4 (Cary, NC, USA), and R 4.0.0 (https://www.R-project.org. A two-sided *p*-value of less than 0.05 was considered significant.)

## 5. Conclusions

The expression of the RelB-BLNK axis could serve as an indicator for selecting patients who would respond to redox-active anticancer agents, thus coupling the novel concept of redox therapy using BET to precision medicine with the goal of improving quality of life after therapy.

## Figures and Tables

**Table 1:** BET as a radioprotector of non-cancer prostate cells (PZ) and a radiosensitizer of PCa cells: PC3, DU145, and C4-2B. α represents single-hit kill and β two-hit kill, which correlate with low-dose killing and high-dose killing.

	All Data	ɑ	β	R Square	D0 Survival (37%)
PZ	Vehicle	−0.148	0.002	0.971	4.3
	1 μM	−0.163	0.004	0.989	4.2
	5 μM	−0.155	0.002	0.992	4.2
	10 μM	−0.115	−0.003	0.99	4.6
	20 μM	−0.114	−0.003	0.977	4.7
PC3	Vehicle	−0.229	0.013	0.997	3.2
	1 μM	−0.266	0.018	0.997	2.9
	5 μM	−0.275	0.02	0.986	2.7
	10 μM	−0.281	0.021	0.991	2.6
	20 μM	−0.254	0.017	0.984	2.8
DU145	Vehicle	−0.239	0.016	0.995	3.2
	1 μM	−0.219	0.013	0.99	3.3
	5 μM	−0.222	0.012	0.996	3.4
	10 μM	−0.231	0.015	0.994	3.3
	20 μM	−0.256	0.018	0.97	2.9
C4-2B	Vehicle	−0.4	0.04	0.991	1.8
	1 μM	−0.433	0.046	0.989	1.6
	5 μM	−0.427	0.045	0.989	1.65
	10 μM	−0.427	0.044	0.991	1.65
	20 μM	−0.429	0.044	0.992	1.65

## Data Availability

Data supporting Figure 1 are in an excel file. Data supporting Figure 5 can be found as described in the text.

## Supplementary Materials

The following supporting information can be downloaded at: https://www.mdpi.com/article/10.3390/ijms23126409/s1.

## Abbreviations

4HNE	4 hydroxynonenal
BET	Betamethasone
BLNK	Adaptor protein of B-cell antigen receptor
C4-2B	LNCaP-C4-2B- LNCaP-derived cell line
EM	Electron microscopy
Gy	Gray, unit of radiation dose
IP	Immunoprecipitation
LNCaP	Androgen-responsive prostate cancer cells
PC3	Androgen-independent prostate cancer cells
PCa	Prostate cancer
PE	Plating efficiency
PEG-CAT	Polyethylene glycol–Catalase
PrEC	Normal human prostate epithelial cells
PZ	Immortalized prostate epithelial PZ-HPV-7
RelB	Rel/NF-kB transcription factors
ROS	Reactive oxygen species
RT	Radiation therapy
