# Supplementary material for: The RelB-BLNK Axis Determines Cellular Response to a Novel Redox-Active Agent Betamethasone during Radiation Therapy in Prostate Cancer
Source: Int J Mol Sci. 2022 Jun 8;23(12):6409. doi: 10.3390/ijms23126409 (PMC9223669; doi:10.3390/ijms23126409)
Supplement: Supplementary file 1 [file ijms-23-06409-s001.zip › ijms-1683649-supplementary.pdf]

Supplementary Figures

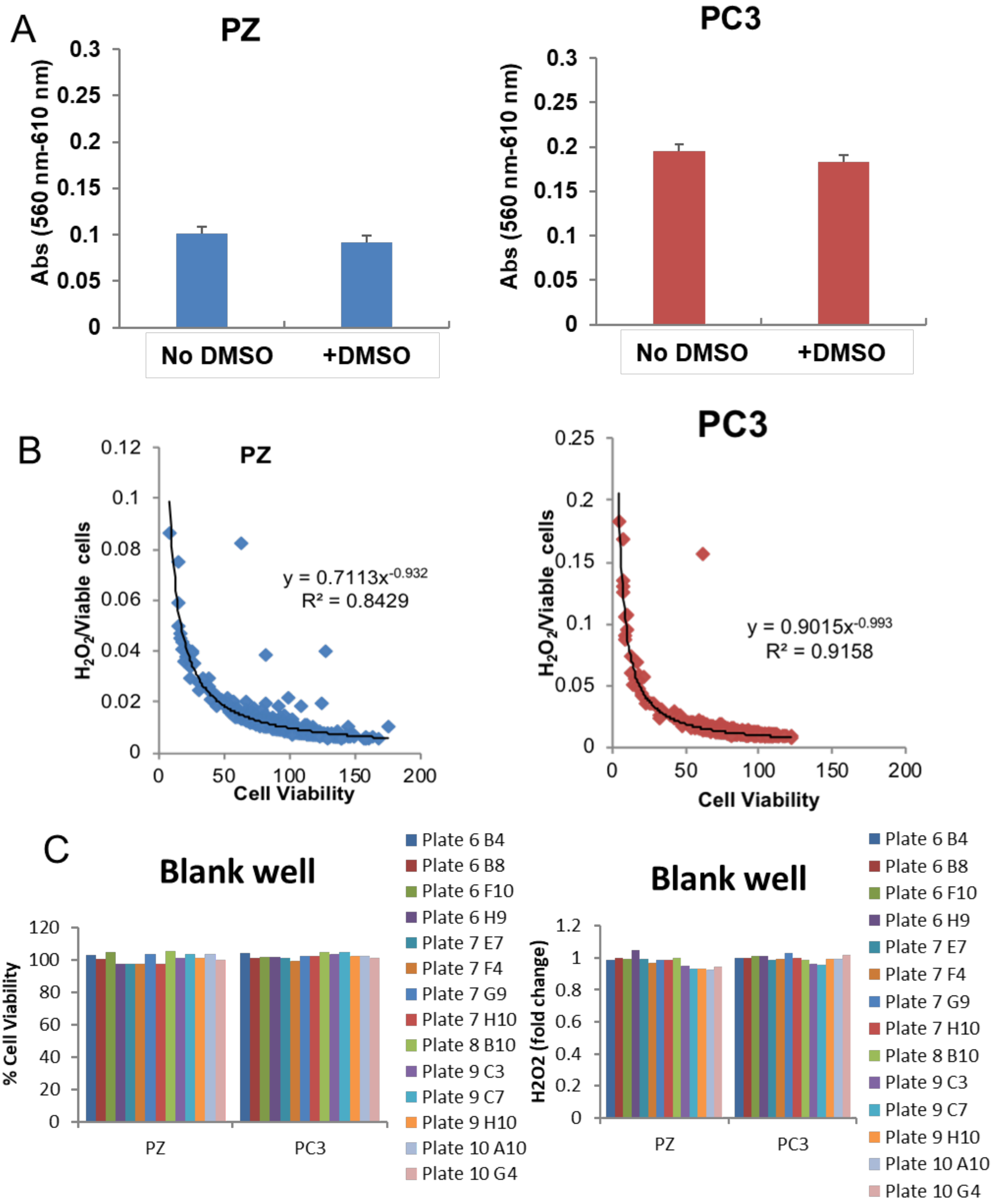

Supplementary Figure S1. High level of H_2O_2 production correlated with less cell viability in both PC3 and PZ cells. (A) Effect of DMSO on cell viability. MTT assay of PC3 cells and PZ cells after treatment with DMSO

for 24 hours. (B) H_2O_2 production per viable cell based on Amplex red assay after treatment of PC3 cells and PZ cells with FDA drugs.

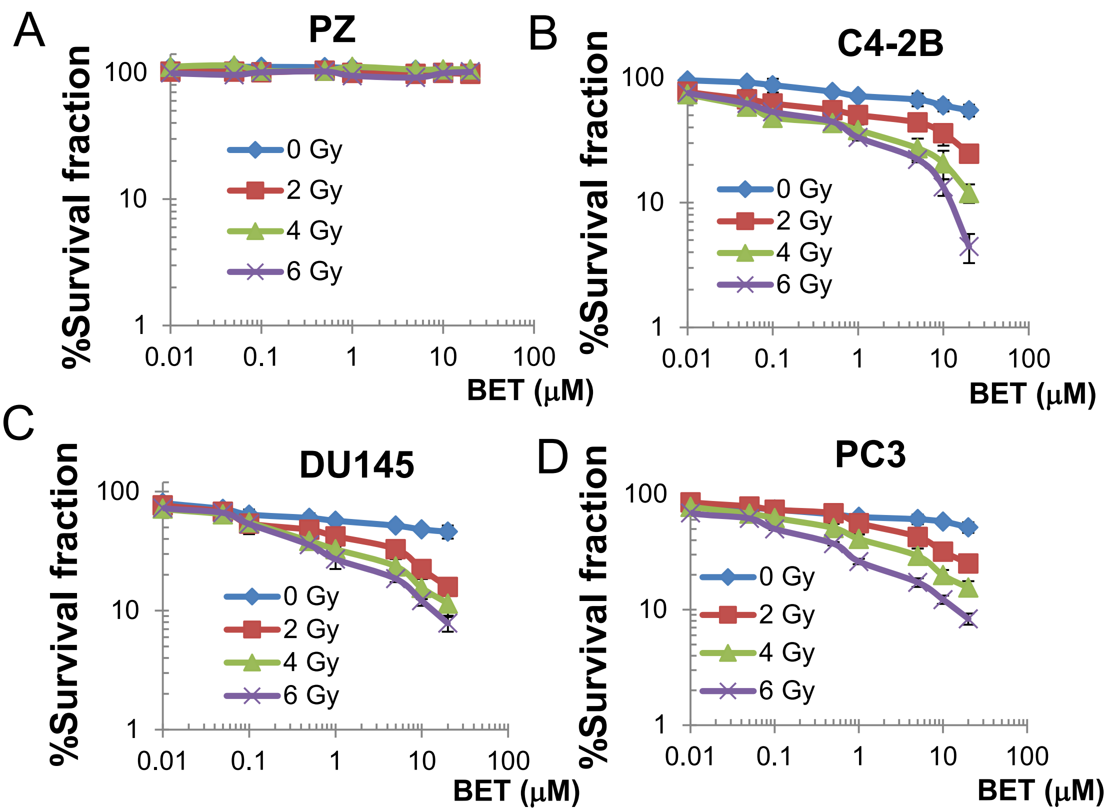

Supplementary Figure S2. Effect of BET as radiosensitizer on prostate cancer cells. Cells were treated with vary doses of BET followed by vary dose of radiation. Survival fraction calculation based on colony formation are performed after 10-12 days.

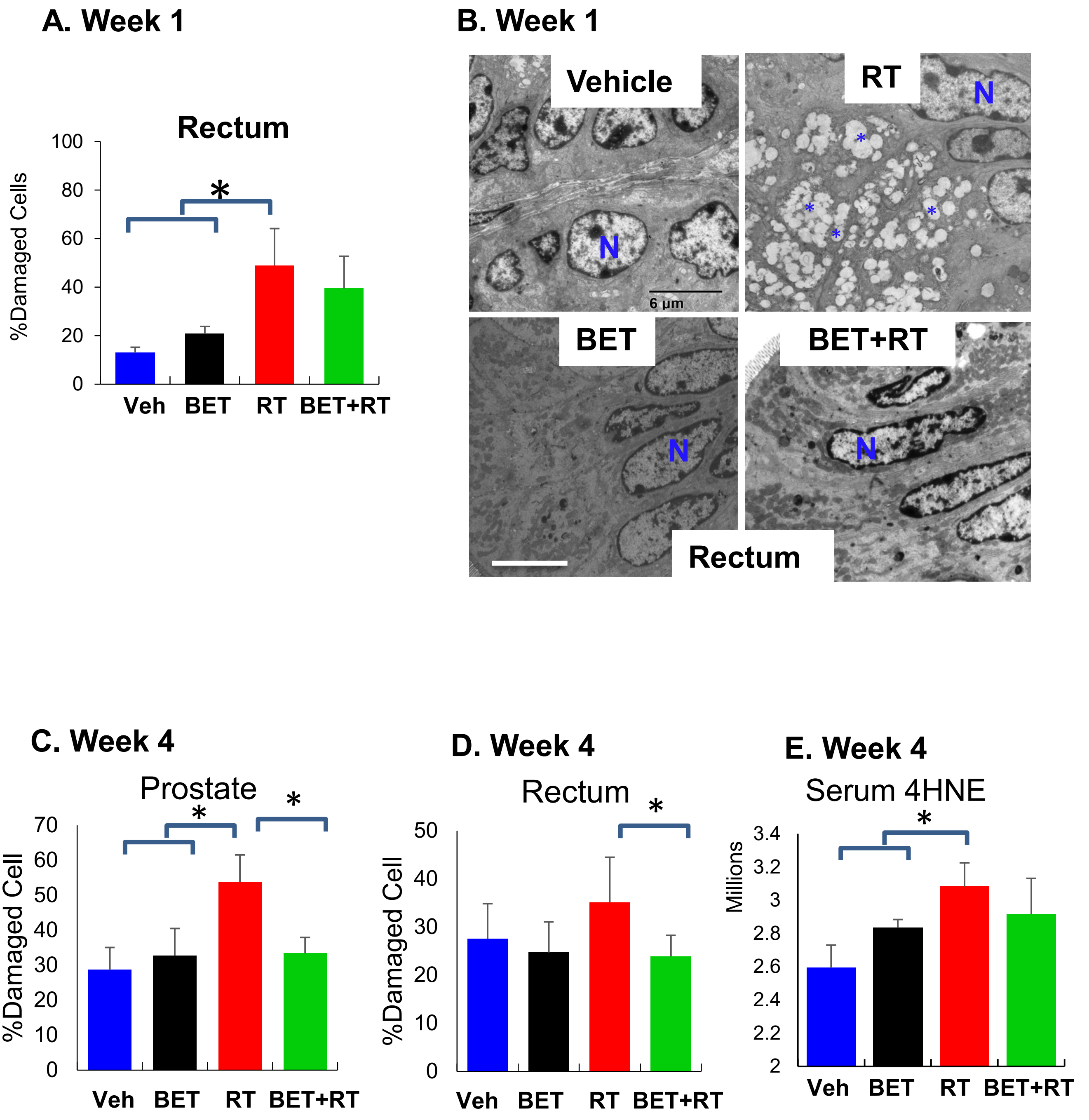

Supplementary Figure S3. **Effect of BET on normal tissues**. BET mitigated prostate damage and rectum damage, which were associated with a decrease in 4HNE protein adduct and PSA level in the serum after treatment with RT (10 Gy) for 4 weeks. (A) % of rectum damage 1 week after RT+ BET treatment. (B) Representative photographs of damaged rectum. N=Nucleus. Star=Damaged mitochondria as indicated by loss of cristae and vacuolization of mitochondria. Bar = 6 µm. (C) % of prostate and (D) rectum damage after treatment for 4 weeks. (E) Serum 4HNE (RU= Relative Arbitrary Unit) after 4 weeks of treatment. *p-value < 0.05.

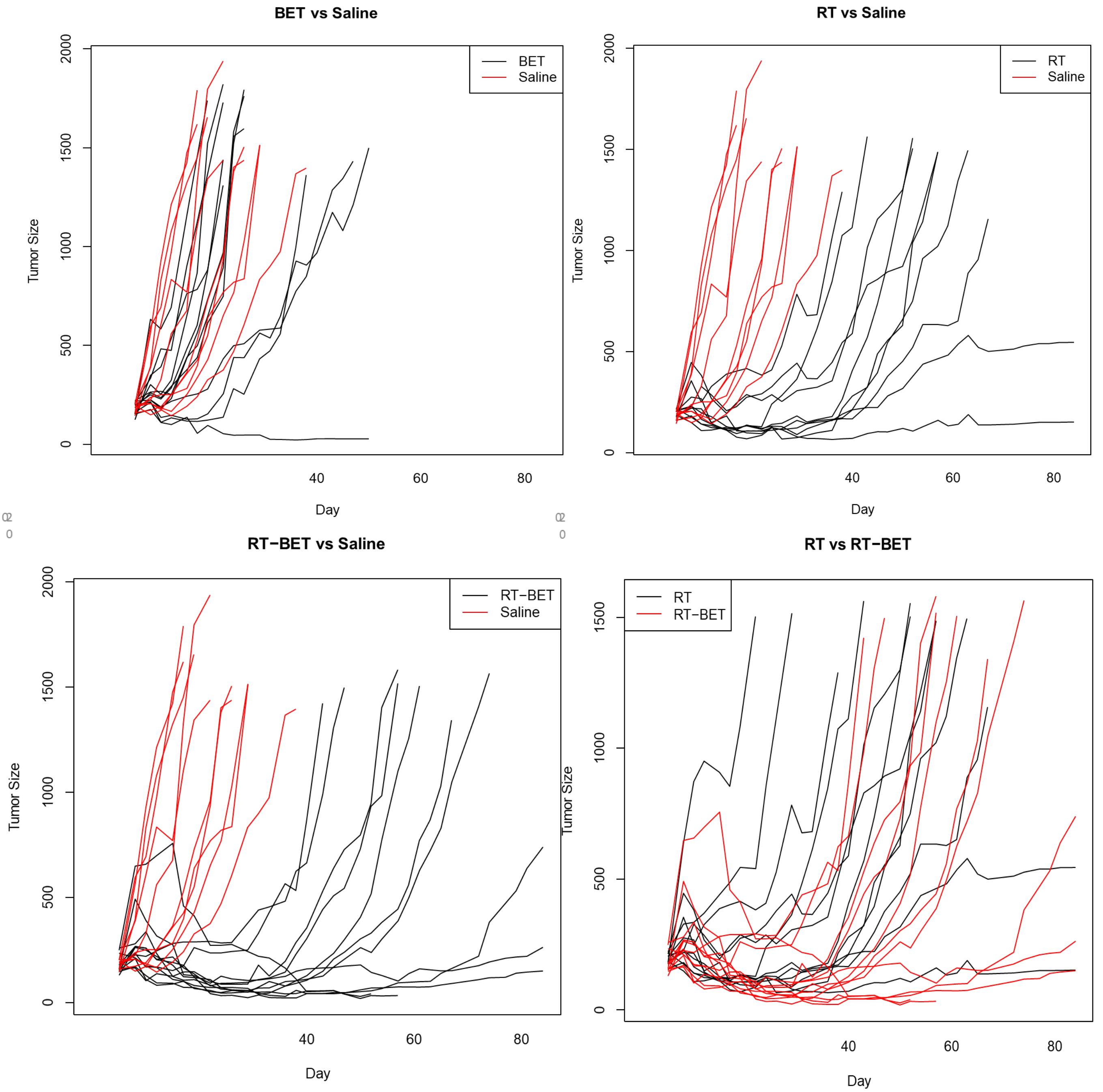

Supplementary Figure S4. **Effect of BET on tumor growth**. BET did not interfere with radiation therapy in tumor bearing mice. Mice were s.c. xenografted with PC3 cells. Once tumor reach ~250 mm^3, mice were treated with BET (20 mg/kg) and RT (2 Gyx5). Tumor size of individual mouse in each group are reported.

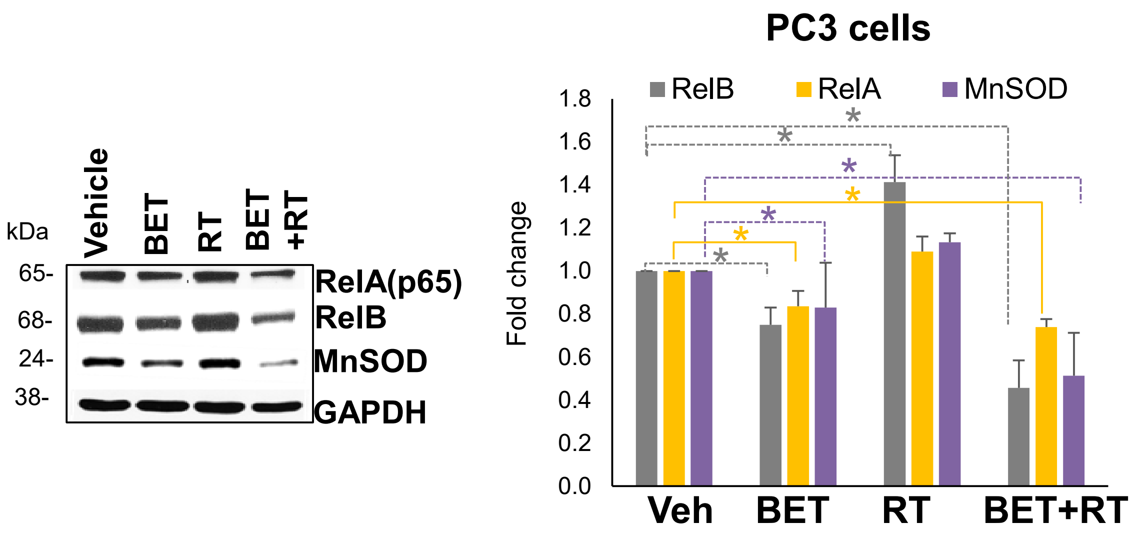

Supplementary Figure S5. BET suppress RelB expression and RelB-associated proteins. Representative western blots and quantitative analysis of protein expression normalize with GAPDH in PC3 cells. N=3. *p-value < 0.05. Three separate experiments were tested.

Pathway analysis

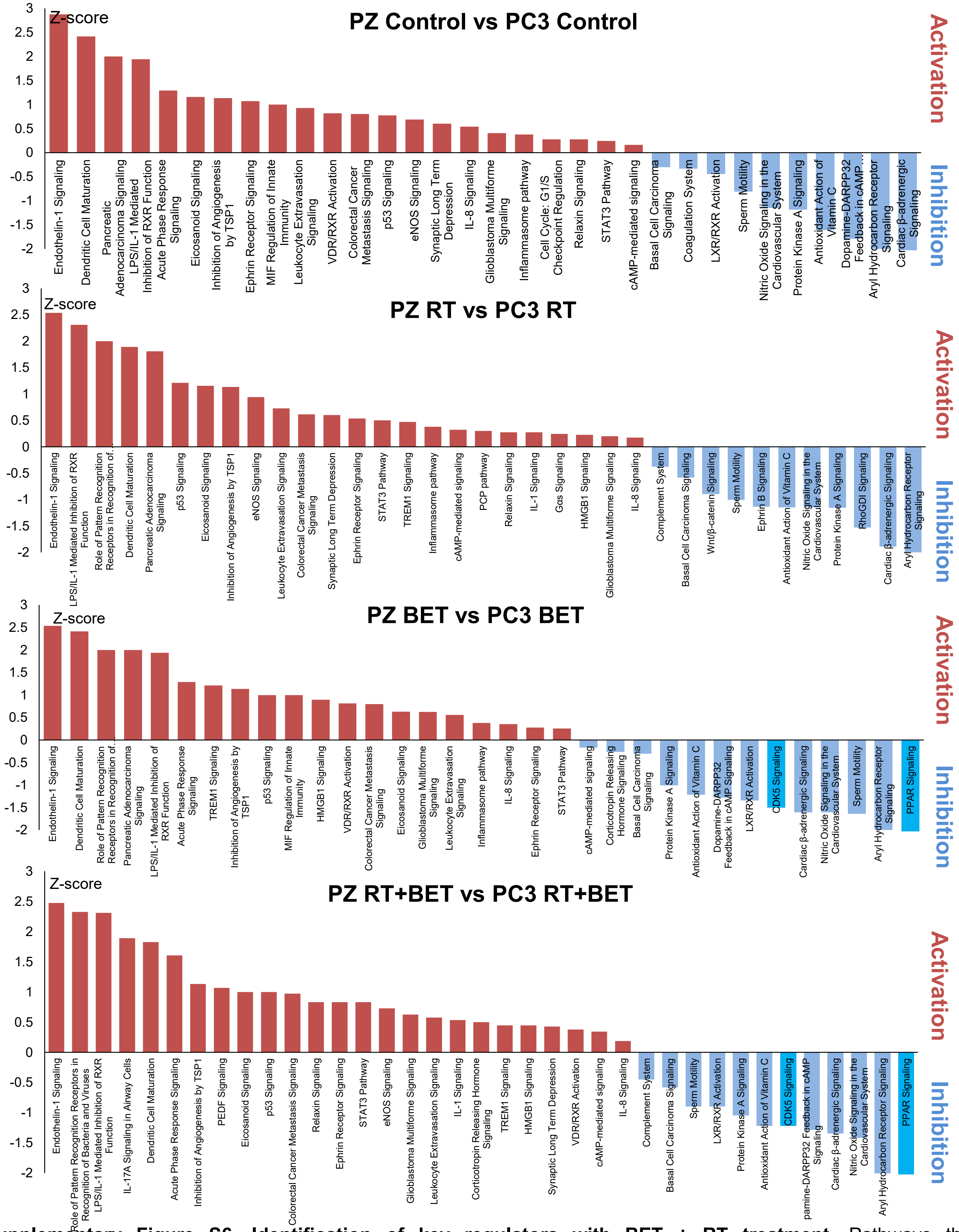

Supplementary Figure S6. Identification of key regulators with BET ± RT treatment. Pathways that were differentially modulated by radiation in the presence of BET (up-regulated [Red] and inhibited [Blue] in PZ cells compared to PC3 cells for each treatment). Data obtained from whole transcriptome RNA Seq analyses of PZ and PC3. The rank order was determined by the indicated IPA z-score (y-axis) which are the predictions of the activation/inhibition states of canonical pathways.

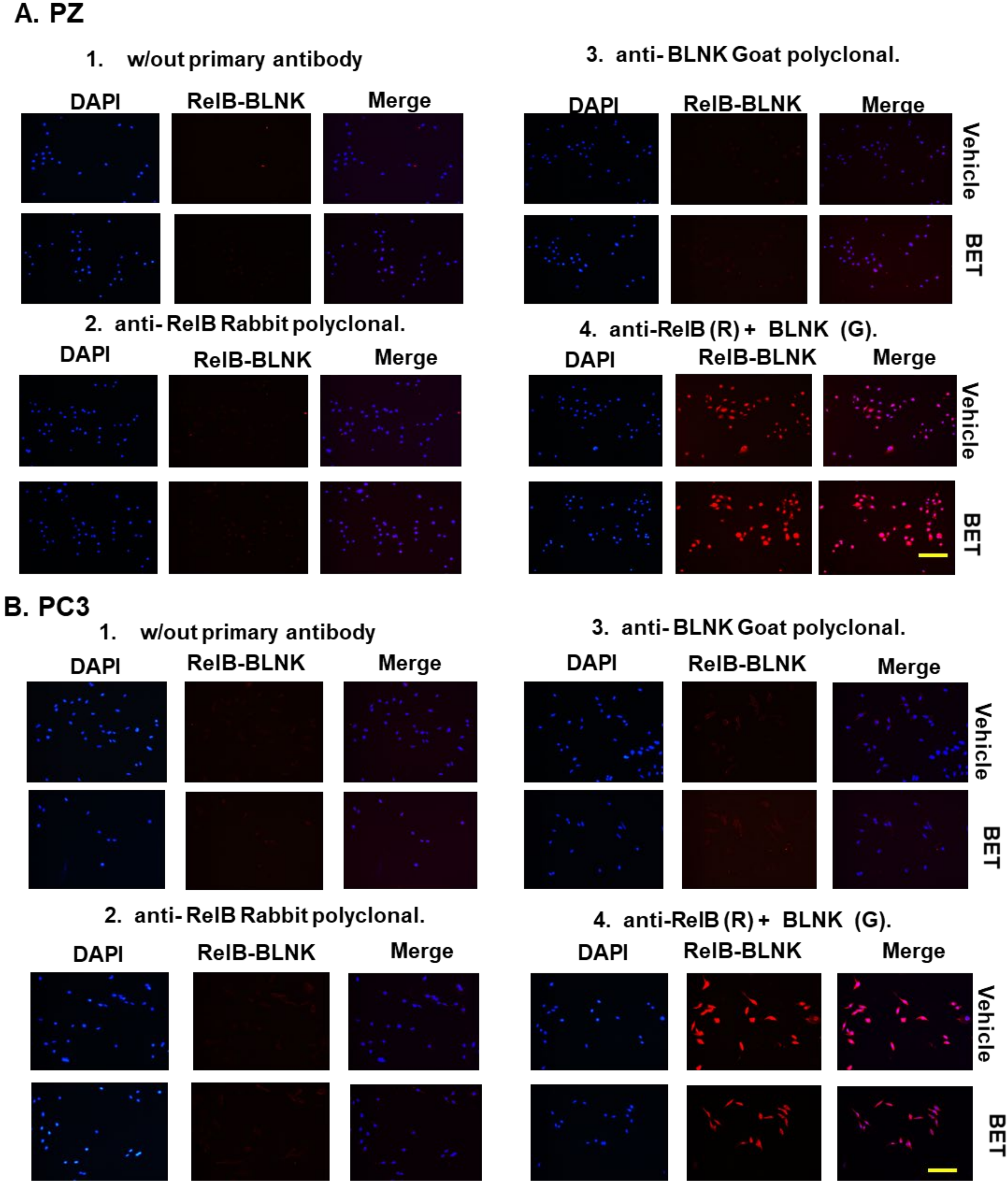

Supplementary Figure S7. PLA ligation assays of (A) PZ cells and (B) PC3 cells after BET treatment for 24 hours. R = Anti-rabbit antibody. G= Anti-goat antibody. Bar = 50 μm.

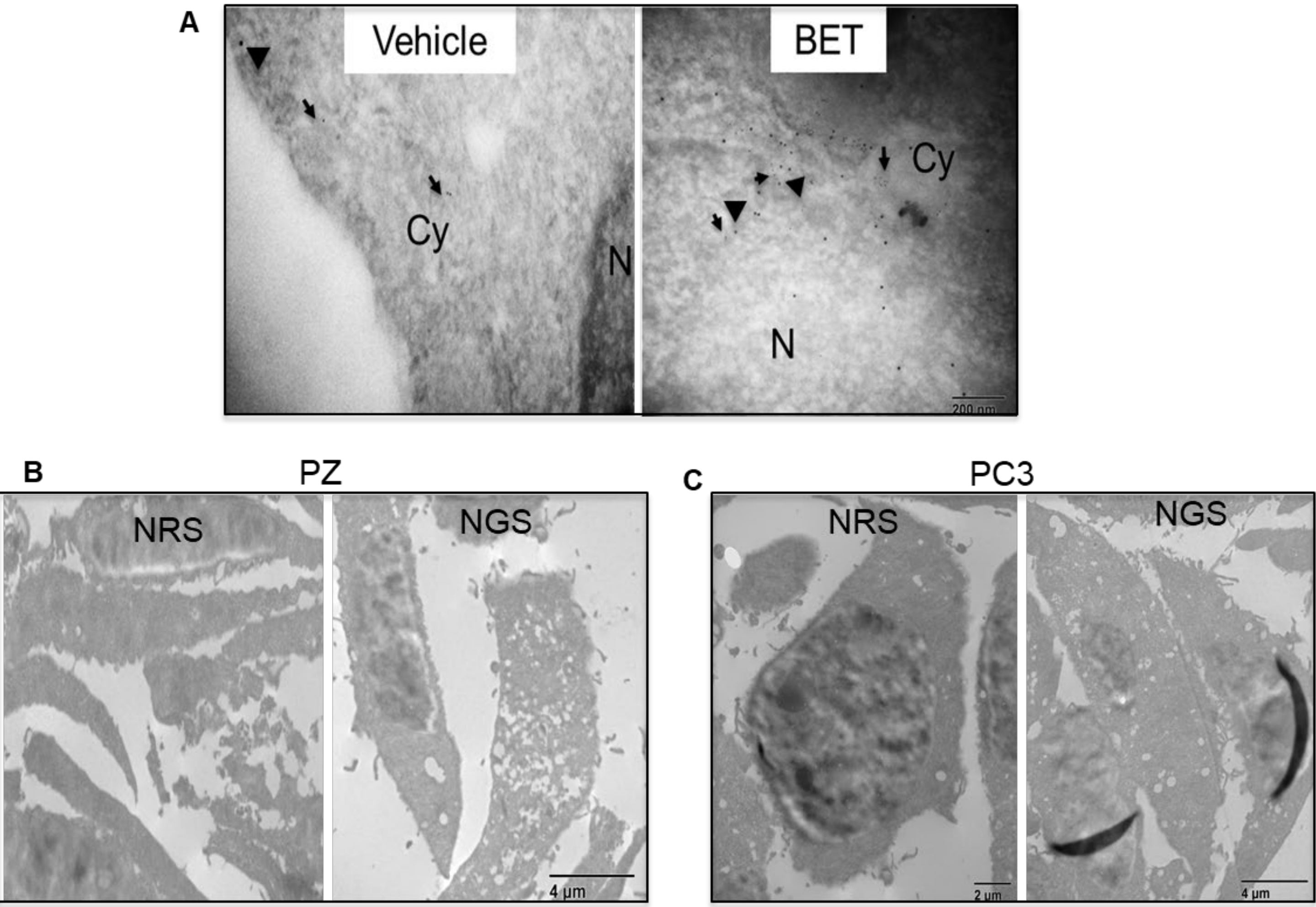

Supplementary Figure S8 Representative electron micrograph. (A) Photograph displays the localization of RelB (6 nm gold beads, arrows) and BLNK (10 nm gold beads, arrow heads) in nucleus and cytoplasm of PZ cells (15000X). RelB and BLNK were found more in the nucleus after BET treatment but were not detectable in the nucleus of vehicle-treated group. (B) PZ cells and (C) PC3 cells stained with the normal rabbit serum (NRS) or normal goat serum (NGS) (6000X). N=Nucleus. Cy=Cytoplasm.

A

siRNA RelB

IDs11917

https://www.thermofisher.com/order/genome-database/details/sirna/s11917?pluginName=&CID=&ICID=

IDs11918

https://www.thermofisher.com/order/genome-database/details/sirna/s11918?CID=&ICID=&subtype=sirna_silencer_select

IDs11919

https://www.thermofisher.com/order/genome-database/details/sirna/s11919?CID=&ICID=&subtype=sirna_silencer_select

siRNA BLNK

IDs26556

https://www.thermofisher.com/order/genome-database/details/sirna/s26556?CID=&ICID=&subtype=

IDs26557

https://www.thermofisher.com/order/genome-database/details/sirna/s26557?CID=&ICID=&subtype=

IDs26558

https://www.thermofisher.com/order/genome-database/details/sirna/s26558?CID=&ICID=&subtype=

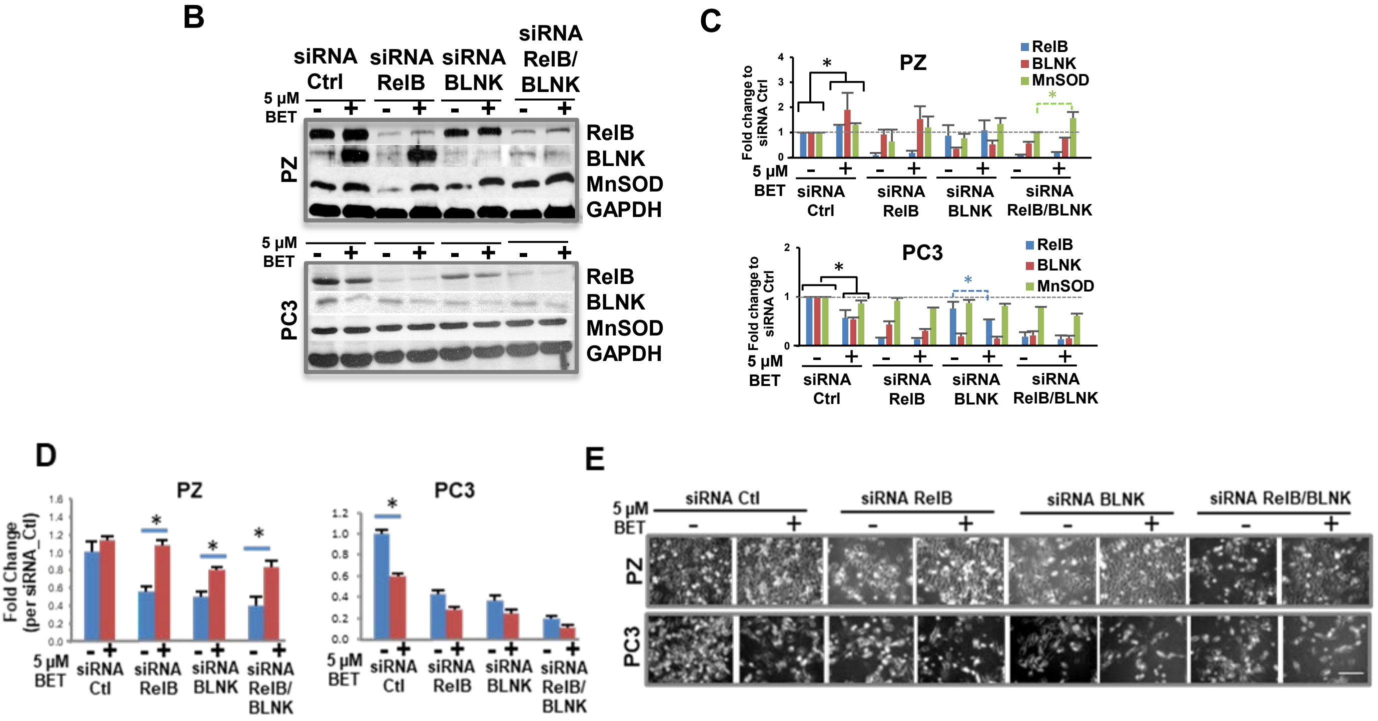

Supplementary Figure S9. A decrease of RelB:BLNK axis with RelB knockdown and BLNK knockdown. Cells were transfected with siRNA for 48 hours and then treated with BET for 24 hours. **(A)** siRNA(s) from Thermo Fisher. (B) Western blot analysis and (C) protein quantification of PC3 and PZ cells. N=3. (D) Trypan Blue assay and (E) represented photograph indicate a decrease in cell killing effect with siRNA against RelB, BLNK, or RelB+BLNK with or without BET treatment. *p-value < 0.05 vs. non-BET. Scale bar = 50 µm.

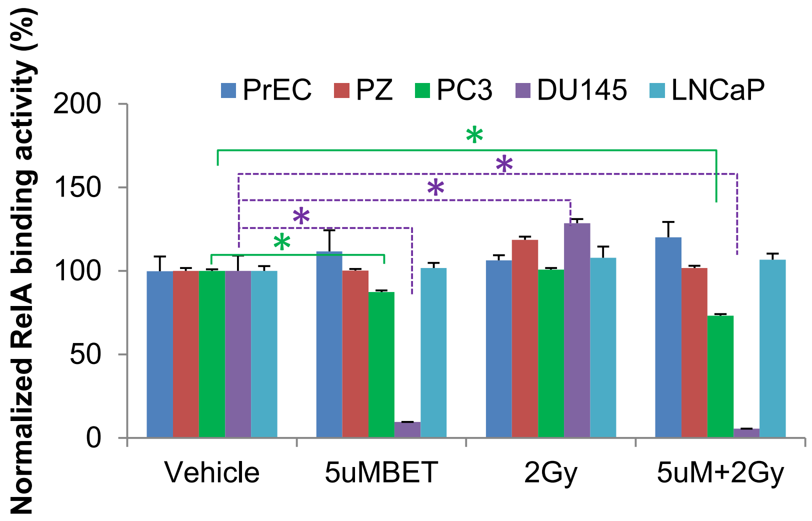

Supplementary Figure S10. RelA activity of normal cells and cancer cells after RT+ BET treatment (24 h) using Active Motif kit. * p-value<0.05.

Supplementary Table S1. Average, high, and low SPRINT scores, for RELB, BLNK, and all predicted interactions

	AVERAGE	HIGH	LOW
Q8WV28	45.93	3919.51	3.67
Q01201	52.50	5207.63	3.67
ALL	81.08	22465.60	3.67

Supplementary Table S2. SPRINT and STRING scores for BLNK and interacting partners from STRING

STRING PPIs	Uniprot id	SPRINT	Coexpression	Experimentally determined	Database annotated	Automated textmining	Combined score
BTK	Q06187	17.1433	0.219	0.883	0.9	0.99	0.999
CD79A	P11912	0	0.23	0.675	0.9	0.982	0.999
CD79B	P40259	13.5255	0.168	0	0.9	0.798	0.981
GRB2	P62993	18.3088	0	0.872	0.9	0.983	0.999
LYN	P07948	137.132	0.09	0.281	0.9	0.786	0.984
PLCG1	P19174	166.236	0.065	0.47	0.9	0.691	0.982
PLCG2	P16885	145.301	0.087	0.47	0.9	0.988	0.999
SH3KBP 1	Q96B97	45.1201	0.062	0.68	0.8	0.944	0.996
SYK	P43405	35.7875	0.18	0.867	0.9	0.985	0.999
VAV1	P15498	38.7099	0.108	0.281	0.8	0.952	0.993
	**Average:**	**61.7264**					

Supplementary Table S3. SPRINT and STRING scores for RELB and interacting partners from STRING

STRING PPIs	Uniprot ID	SPRINT	Coexpression	Experimentally determined	Database annotated	Automated textmining	Combined score
AHR	P35869	82.9184	0	0	0	0.988	0.989
CD40	P25942	62.0186	0.092	0	0	0.987	0.988
CHUK	O15111	234.263	0.054	0.164	0.9	0.983	0.998
HDAC4	P56524	241.772	0	0	0	0.955	0.955
MAP3K14	Q99558	105.674	0.072	0.07	0.9	0.58	0.958
NFKB1	P19838	0	0.247	0.985	0.9	0.985	0.999
NFKB2	Q00653	0	0.795	0.977	0.9	0.99	0.999
NFKBIA	P25963	272.226	0.161	0.786	0.54	0.972	0.997
NFKBIE	O00221	298.834	0.299	0.707	0	0.793	0.954
RELA	Q04206	269.459	0.121	0.871	0.8	0.991	0.979
	**Average:**	**156.717**					

Supplementary Table S4. PPI Contacts between BLNK and RELB that appear 3 or more times out of ten models

BLNK-RELB contact	# of times contact appears				
HIS431-VAL388	4	MOE model 1	MOE model 2	MOE model 4	MOE model 5
HIS431-ARG136	4	MOE model 1	MOE model 2	MOE model 4	MOE model 5
CYS343-TRP189	4	MOE model 3	MOE model 4	MOE model 5	zdock model 2
GLN430-GLU290	4	MOE model 1	MOE model 4	MOE model 5	zdock model 1
HIS431-ILE129	3	zdock model 1	zdock model 2	zdock model 4	
TYR451-HIS191	3	zdock model 1	zdock model 3	zdock model 4	
TYR451-GLU290	3	MOE model 4	MOE model 5	zdock model 3	
ALA452-HIS191	3	zdock model 1	zdock model 3	zdock model 4	
HIS431-VAL128	3	zdock model 1	zdock model 2	zdock model 4	
ARG448-ASP285	3	zdock model 1	zdock model 4	zdock model 5	
HIS431-VAL287	3	zdock model 1	zdock model 2	zdock model 4	
ALA452-TRP189	3	zdock model 1	zdock model 3	zdock model 4	
ARG427-GLU290	3	MOE model 1	zdock model 1	zdock model 4	
GLN430-ARG136	3	MOE model 1	MOE model 4	MOE model 5	
LYS450-ASP285	3	zdock model 1	zdock model 2	zdock model 4	
TYR451-TRP189	3	zdock model 1	zdock model 3	zdock model 4	
ARG427-GLU155	3	MOE model 2	zdock model 1	zdock model 4	
HIS431-CYS389	3	MOE model 1	MOE model 4	MOE model 5	
HIS431-LEU127	3	zdock model 1	zdock model 2	zdock model 4	
PRO345-ARG267	3	MOE model 4	MOE model 5	zdock model 4	
LYS344-LYS187	3	MOE model 4	MOE model 5	zdock model 4	
GLN430-GLN135	3	MOE model 1	MOE model 4	MOE model 5	
LYS450-GLU155	3	MOE model 1	MOE model 4	MOE model 5	
LYS450-HIS191	3	zdock model 1	zdock model 3	zdock model 4	
GLN430-GLU155	3	MOE model 1	MOE model 4	MOE model 5	

Supplementary Table S5. Ranking from docking analysis using DB00443, known active/inactive compounds from PubChem and a background of approved drugs.

PUBCHEM_CID	ACTIVITY_OUTCOME	RANK
155511564	Inactive	25.48%
154659617	Inactive	11.74%
155552172	Inactive	31.21%
155553313	Inactive	31.81%
155556293	Inactive	23.40%
154659619	Active	7.91%
DB00443	presumed active	20.10%
